# Evolutionary analysis of polyproline motifs in *Escherichia coli* reveals their regulatory role in translation

**DOI:** 10.1371/journal.pcbi.1005987

**Published:** 2018-02-01

**Authors:** Fei Qi, Magdalena Motz, Kirsten Jung, Jürgen Lassak, Dmitrij Frishman

**Affiliations:** 1 Department of Bioinformatics, Wissenschaftzentrum Weihenstephan, Technische Universität München, Freising, Germany; 2 Center for Integrated Protein Science Munich, Ludwig-Maximilians-Universität München, Munich, Germany; 3 Department of Biology I, Microbiology, Ludwig-Maximilians-Universität München, Martinsried, Germany; 4 St Petersburg State Polytechnic University, St Petersburg, Russia; University of Oxford, UNITED KINGDOM

## Abstract

Translation of consecutive prolines causes ribosome stalling, which is alleviated but cannot be fully compensated by the elongation factor P. However, the presence of polyproline motifs in about one third of the *E*. *coli* proteins underlines their potential functional importance, which remains largely unexplored. We conducted an evolutionary analysis of polyproline motifs in the proteomes of 43 *E*. *coli* strains and found evidence of evolutionary selection against translational stalling, which is especially pronounced in proteins with high translational efficiency. Against the overall trend of polyproline motif loss in evolution, we observed their enrichment in the vicinity of translational start sites, in the inter-domain regions of multi-domain proteins, and downstream of transmembrane helices. Our analysis demonstrates that the time gain caused by ribosome pausing at polyproline motifs might be advantageous in protein regions bracketing domains and transmembrane helices. Polyproline motifs might therefore be crucial for co-translational folding and membrane insertion.

## Author summary

Polyproline motifs induce ribosome stalling during translation, but the functional significance of this effect remains unclear. Our evolutionary analysis of polyproline motifs reveals that they are disfavored in *E*. *coli* proteomes as a consequence of the reduced translation efficiency, supporting the conjecture that translation efficiency-based evolutionary pressure shapes protein sequences. Enrichment of polyproline motifs in the protein regions bracketing structural domains and transmembrane helices indicates their regulatory role in co-translational protein folding and transmembrane helix insertion. Polyproline motifs could thus serve as protein-level *cis*-acting elements, which directly regulate the rate of translation elongation.

## Introduction

Ribosomes facilitate the synthesis of proteins by translating the nucleotide sequence from an mRNA template. The speed of mRNA translation significantly varies and strongly depends on the amino acids to be incorporated into the growing polypeptide chain [1]. Especially slow is the incorporation of proline [2–4]. The pyrrolidine ring gives proline an exceptional conformational rigidity compared to all other amino acids and makes it not only a poor A-site peptidyl acceptor [2], but also a poor P-site peptidyl donor [3,4]. Translation of two and more consecutive prolines dramatically impairs the peptidyl transfer reaction and eventually causes ribosomes to stall [3,5–9]. Although basically all diproline comprising motifs cause translational stalling [5,10], the arrest strength is influenced by physical and chemical properties of the adjacent amino acids that affect the conformation of the nascent polypeptide chain. Based on proteomic approaches combined with systematic *in vivo* and *in vitro* analyses, a hierarchy of arrest peptides was described [5,9–11]. Thereby triplets such as PPP, D/PP/D, PPW, APP, G/PP/G and PPN cause strong ribosome stalling whereas *e*.*g*. L/PP/L, CPP or HPP result in a rather weak translational pause. Moreover, the stalling strength is modulated by amino acids located—up to position -5—upstream of the arrest motif [10,12,13]. In this respect, H, K, Q, R or W further pronounce the arrest whereas C, G, L, S or T attenuate it. We therefore define a “polyproline motif” as a consecutive stretch of prolines with flanking residues: X_(-2)_X_(-1)_-nP-X_(+1)_, n≥2; where X_(-2)_, X_(-1)_ and X_(+1)_ can be any amino acid.

Regardless of the difficulties to translate consecutive proline coding sequences, they occur frequently within prokaryotic and eukaryotic proteomes [14,15]. This in turn implies that the benefits of retaining polyproline motifs significantly outweigh their costs to incorporate them into the nascent polypeptide chain [16]. Proline is unique in terms of being the sole amino acid to adopt *cis* and *trans* conformations, both of which are nearly energetically equal and naturally occur in proteins [17–19]. Notably, a sequence of consecutive prolines results in the formation of either the right-handed poly (*cis-*) proline helix I (PPI) or the left-handed poly (*trans*-) proline helix II (PPII). Beside α-helix and β-sheet, PPII helix is considered to be the third major secondary structure element in proteins and plays an important role in mediating protein-protein and protein-nucleic acid interactions [20–23]. Three consecutive prolines are also an integral part of the active center in the universally conserved Val-tRNA synthetase ValS [14]. The proline triplet in ValS is essential for efficient charging of the tRNA with valine and for preventing mischarging by threonine. These two examples illustrate why nature has evolved a specialized translation elongation factor, referred to as EF-P in bacteria or e/aIF-5A in eukaryotes and archaea, to alleviate ribosome stalling at polyproline motifs [3,5–9,16,24]. The importance of polyproline motifs in proteins is further underlined by the fact that *efp* mutants are characterized by pleiotropic defects. Reportedly, the absence of EF-P impairs bacterial fitness [25,26], membrane integrity [27], motility [28], antibiotic sensitivity [29] and is ultimately lethal for certain bacteria such as *Mycobacterium tuberculosis* [30] and *Neisseria meningitides* [31]. Similarly, IF-5A is an essential protein in archaea [32] as well as in eukaryotes [33] where eIF-5A is associated *e*.*g*. with cancer [34] and HIV infection [35].

EF-P alleviates polyproline motif-dependent translational arrest, but does not prevent ribosome pausing at these sequences [6,10,36]. The fact that polyproline motifs form a functionally important structural element—the PPII helix—and at the same time interfere with translation poses a major evolutionary conundrum. Are polyproline motifs disfavored during evolution due to their translational burden? Does ribosome stalling caused by polyproline motifs regulate the speed of translation at the protein level in the same way as rare genetic codons and RNA secondary structures cause translational pause at the RNA level [37,38]? To address these questions, we conducted an evolutionary analysis of polyproline motifs in the proteomes of 43 *E*. *coli* strains. Our analysis revealed evolutionary selection against polyproline motifs as a consequence of the reduced translation efficiency. Against the overall background of polyproline motif depletion, we observed their frequent occurrence in the vicinity of translational start sites, in the inter-domain regions of multi-domain proteins, and downstream of transmembrane helices, where slow-translating codons are also enriched. This indicates the potential involvement of polyproline motifs in co-translational protein folding and transmembrane helix insertion.

## Results

### Polyproline motifs are underrepresented in *E*. *coli* proteomes

**Fig 1:**
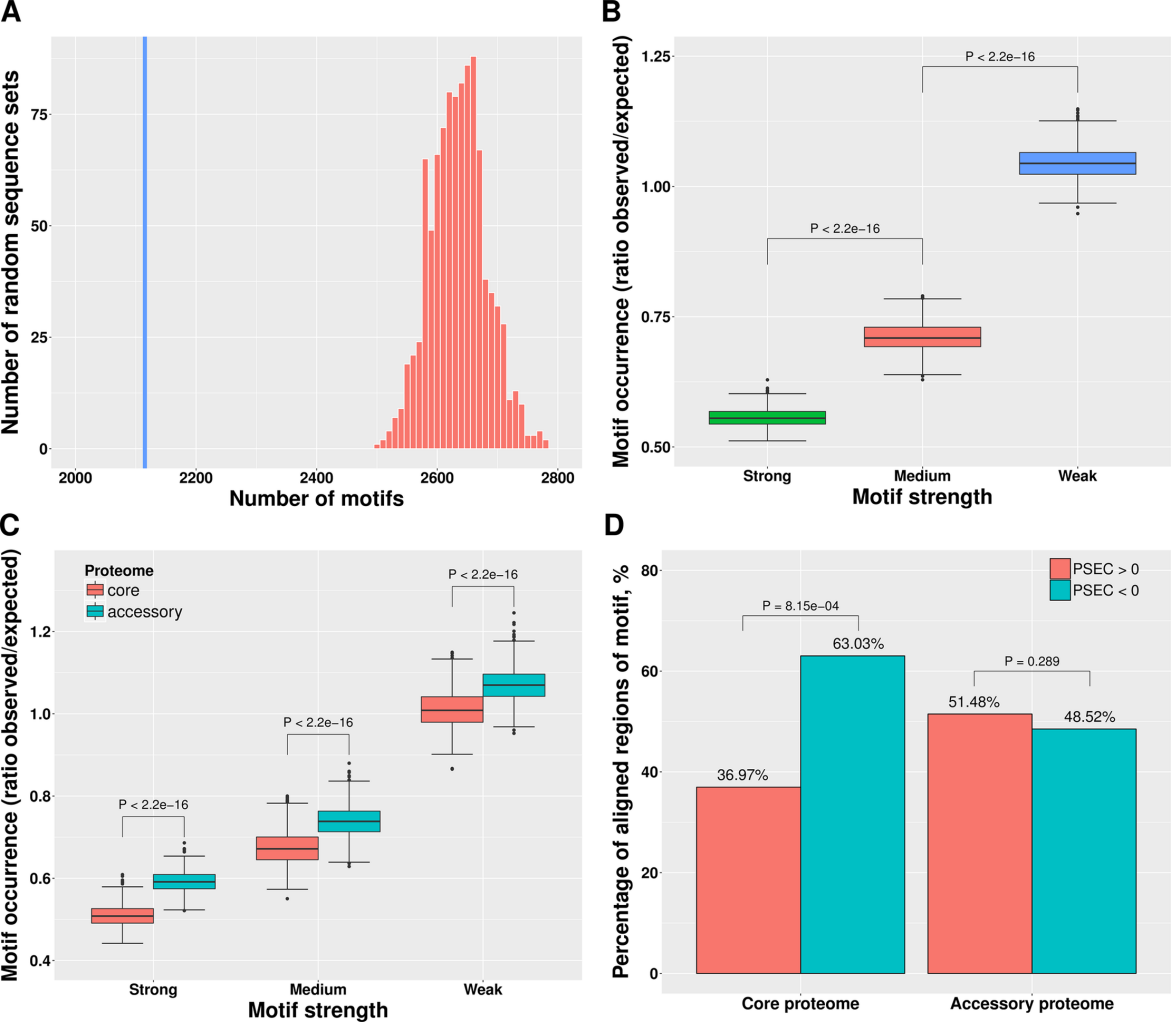
Distribution and conservation of polyproline motifs. (A) Occurrence of polyproline motifs in *E*. *coli* K-12 MG1655 is lower than the random level (fold change 0.80). The histogram shows the numbers of motifs found in 1,000 sets of random sequences, and the blue line shows the number of motifs found in real sequences. (B) Numbers of polyproline motifs negatively correlate with the strength of the ribosome stalling effect in *E*. *coli* K-12 MG1655. The differences are significant according to Mann-Whitney-Wilcoxon test. (C) Occurrence of polyproline motifs in the core proteome of *E*. *coli* K-12 MG1655 is lower than that in the accessory proteome. The differences are significant according to Mann-Whitney-Wilcoxon test. (D) In the core proteome more aligned regions have a negative PSEC (chi-squared test) while in the accessory proteome PSEC values display no strong preference.

We first investigated the overall frequency of polyproline motifs in *E*. *coli* strains and found 99,386 polyproline motifs within 68,710 (33.3%) proteins from the 43 proteomes considered in this study. Out of these 68,710 proteins, 47,056 proteins (68.5%) harbor only one polyproline motif, 15,027 proteins (21.9%) have two polyproline motifs, and 6,627 proteins (9.6%) have more than 2 polyproline motifs (S1 Fig). We identified 22,253 (22.4%), 21,953 (22.1%) and 55,149 (55.5%) polyproline motifs with strong, medium and weak ribosome stalling effect, respectively. We found that polyproline motifs are significantly underrepresented in all the 43 *E*. *coli* proteomes compared with randomly generated protein sequences (Fig 1A and S2 Fig; *p*-values < 2.2e-16; average fold change 0.82). Pairs of consecutive prolines show the lowest ratio between the observed and expected frequency (0.84) compared to all other pairs of identical amino acids in *E*. *coli* K-12 MG1655 (the ratios of the other amino acids are between 1.00 and 1.66, with a mean of 1.17). Moreover, normalized by the random level, the numbers of polyproline motifs negatively depend on the strength of the ribosome pausing effect in all *E*. *coli* proteomes: in *E*. *coli* K-12 MG1655, for example, polyproline motifs with strong, medium and weak ribosome stalling effect constitute 55.5%, 70.9% and 104.4% of the random level, respectively (Fig 1B and S3 Fig). Collectively, these findings suggest the existence of evolutionary pressure against ribosome pausing.

To investigate this hypothesis, we grouped the proteins into the core proteome, which encompasses conserved, evolutionary older sequences, and the accessory proteome, which mainly contains proteins of younger origin. Assuming that evolution disfavors polyproline motifs, one would expect them to occur less frequently in the core proteome. Indeed, significantly fewer polyproline motifs were found in proteins belonging to the *E*. *coli* core set, independent of the arrest strength (Fig 1C and S4 Fig; Mann-Whitney-Wilcoxon test, *p*-values < 2.2e-16; the ratios between motif occurrence of core and accessory proteomes for strong, medium and weak strengths are 0.88, 0.87 and 0.93, respectively). We note that we cannot fully rule out the possibility that this observation is partly due to the differences between the core and accessory proteomes in terms of their functional repertoire (GO terms) and gene expression levels (mean of log_10_ value: translation efficiency, 1.68 vs 1.57; protein abundance, 2.05 vs 1.86).

We also compared the occurrence of polyproline motifs across the 43 *E*. *coli* strains. We found that they have the highest occurrence in strain O157:H7 (fold change 0.85) and the lowest occurrence in strain UM146 (fold change 0.79). As seen in S5 Fig, the polyproline motif occurrence in the core proteomes of *E*. *coli* strains is quite similar (mean fold change 0.76, standard deviation 0.003), while the accessory proteomes are more diverse in this respect (mean fold change 0.85, standard deviation 0.023). We also found that polyproline motif occurrence is highly correlated with the number of proteins in proteomes (S6 Fig; Pearson’s r = 0.68, *p*-value = 4.82e-7). By definition, core proteome sizes of *E*. *coli* strains are the same, while accessory proteome sizes differ. Therefore, this result actually indicates that strains with larger accessory proteomes have more polyproline motifs, as already discussed above.

### Variation of ribosome stalling strength in *E*. *coli* evolution

We next investigated changes in ribosome stalling strength caused by polyproline motifs in the *E*. *coli* proteins by considering 3,280 orthologous groups with at least 3 proteins and at least one polyproline motif. Within these orthologous groups, we identified 4,980 aligned regions containing polyproline motifs, of which 1,568 showed changes of the ribosome stalling effect states. Out of the 1,923 evolutionary events 955 were gain events (change from a weaker or no stalling effect state to a stronger state) and 968 were loss events (change from a stronger stalling effect state to a weaker or no stalling effect state). The propensity for stalling effect change (PSEC) was calculated for each of these aligned regions as described in the *Materials and Methods* section. In the core proteome, substantially more aligned regions displayed a negative PSEC (Fig 1D; 63.03% vs 36.97%; chi-squared test, *p*-value = 8.15e-4), indicating that the ribosome stalling effect tends to be lost in evolution. In line with this finding, in the phylogenetically younger accessory proteome, PSEC still displayed no strong preference with 51.48% and 48.52% aligned regions possessing positive and negative PSEC, respectively (Fig 1D; chi-squared test, *p*-value = 0.289). These results are also in line with the notion that evolution generally disfavors polyproline motifs in *E*. *coli*.

### Translational efficiency is the evolutionary driving force for selecting against polyproline motifs

**Fig 2:**
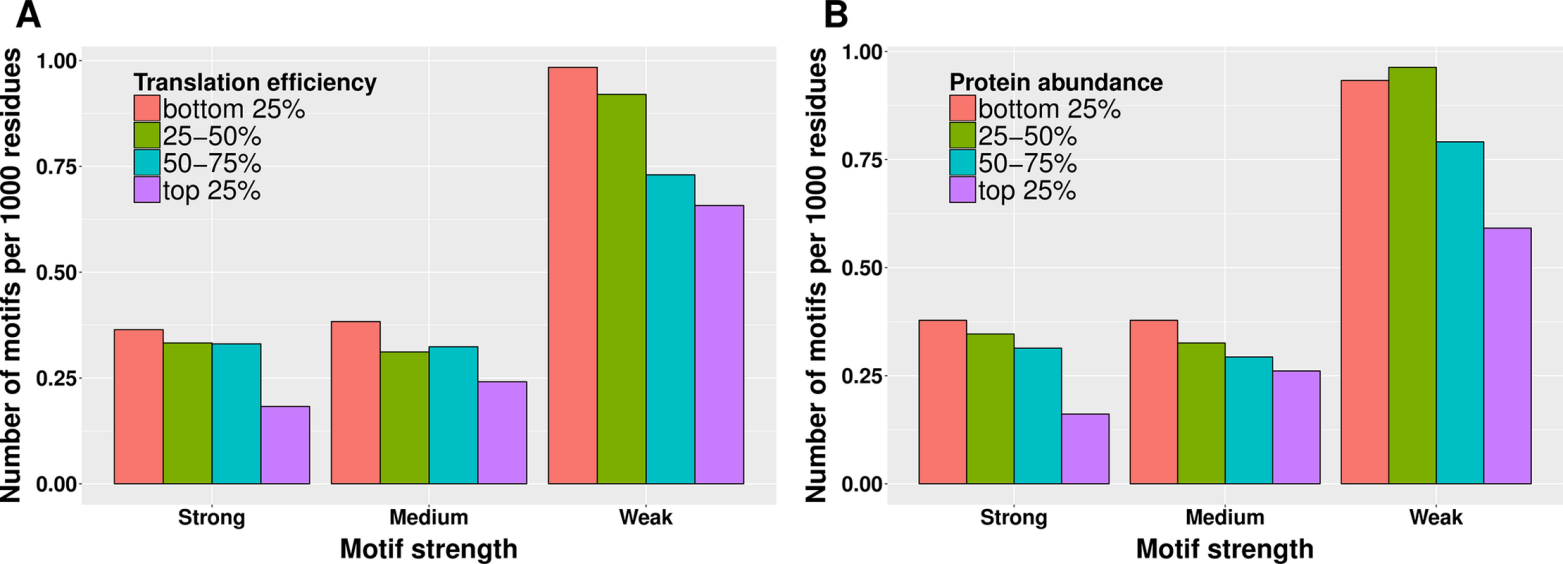
Correlation between translation efficiency, protein abundance and frequency of polyproline motifs. (A) Proteins with high translation efficiency tend to have fewer polyproline motifs (Spearman's rho = -0.105, *p*-value = 1.13e-5). (B) High abundance proteins tend to have fewer motifs (Spearman's rho = -0.135, *p*-value = 1.47e-8).

The efficiency of translation and consequently biosynthesis correlates with both translation initiation and elongation rates [39]. Translation elongation rate in turn depends on multiple factors, such as codon bias [36], tRNA levels [40] and the amino acid to be incorporated [2,41], but can also be influenced by an amino acid sequence such as consecutive prolines [5,7,10]. Accordingly, we investigated whether there is a connection between the relative frequency of polyproline motifs and translational efficiency in *E*. *coli* K-12 MG1655, and found that they are negatively correlated (Fig 2A; Spearman’s rho = -0.105, *p*-value = 1.13e-5), which is especially evident in the top 25% of most efficiently translated proteins and for polyproline motifs known to cause a strong translational pause. Occurrence of polyproline motifs also anticorrelates with relative protein abundance (Fig 2B; Spearman’s rho = -0.135, *p*-value = 1.47e-8). Thus, in the course of evolution polyproline motifs are more disfavored in those proteins that have a high copy number per cell and need to be efficiently translated, implying a translation efficiency-driven selection pressure against polyproline motifs.

### Polyproline motifs as regulatory elements in protein synthesis

**Fig 3:**
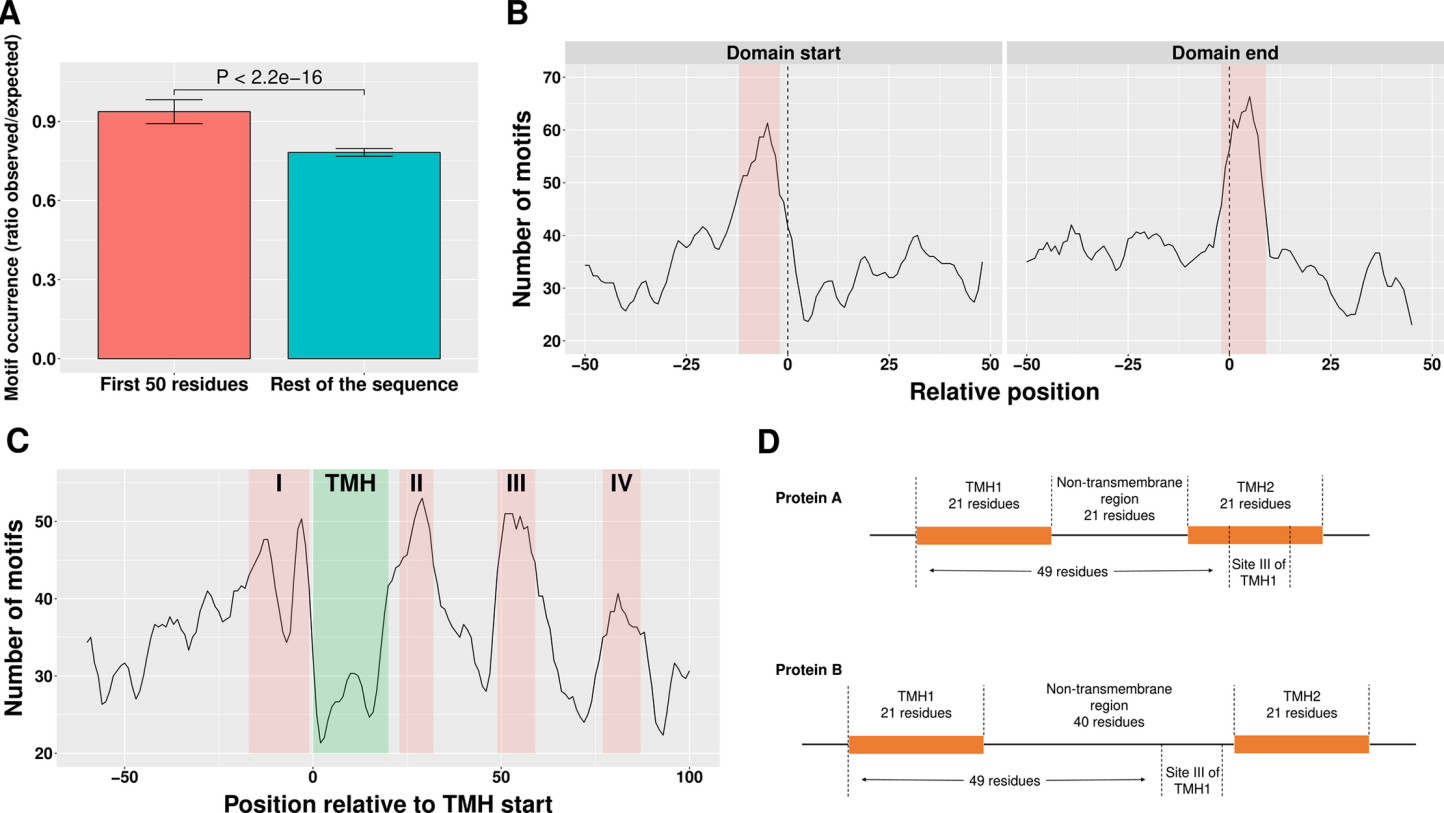
Functional role of polyproline motifs. (A) Occurrence of polyproline motifs in the first 50 residues is higher than elsewhere in the protein sequence (Mann-Whitney-Wilcoxon test, *p*-value < 2.2e-16; fold change 0.94 vs 0.78). Error bars indicate the standard deviation. (B) Occurrence of polyproline motifs is associated with domain boundaries. Regions with relatively high motif occurrence are marked red. Data are smoothed over a three-residue window. Left: frequency of motifs relative to domain start (dashed line). Right: frequency of motifs relative to domain end (dashed line). The enrichment of motifs in these two regions is significant (*p*-values < 0.05; fold changes 1.19 and 1.23). (C) Frequency of polyproline motifs relative to the start position of TMH. TMH is marked green (assuming the typical length of 21 residues). Regions with high motif frequency are marked red. Data are smoothed over a three-residue window. (D) Schematic illustration of the site III location relative to TMH and the non-transmembrane region. In protein A site III of TMH1 locates in the TMH2 while in protein B site III of TMH1 is in the non-transmembrane region.

We next investigated whether polyproline-mediated ribosome pausing is exploited in the regulation of translation, focusing on the reference strain *E*. *coli* K-12 MG1655 comprising 2,115 polyproline motifs in 1,477 proteins (33.9% of the whole proteome) (S1 Table). In 2010, Tuller *et al*. discovered reduced translation efficiency within the first 50 codons of the coding regions [42]. The authors suggested that a slow ramp at the beginning of the ORF might serve as a late stage of translation initiation, being a probate means to reduce ribosomal traffic jams in order to minimize the cost of protein biosynthesis [42–44]. Multiple factors, including slow-translating codons, strong mRNA structures and positively charged amino acids, were implicated in the formation of the ramp [43,44]. We were therefore curious whether there exists an enrichment of polyproline motifs around the start sites of *E*. *coli* K-12 MG1655 proteins. In the 2,115 polyproline motifs of *E*. *coli* K-12 MG1655, 325 were found in the first 50 amino acids, and 1,771 located elsewhere in the protein sequence. After normalization by random level, we found a clear enrichment of polyproline motifs in the N-terminal 50 residues (Fig 3A; Mann-Whitney-Wilcoxon test, *p*-value < 2.2e-16; fold change 0.94 vs 0.78). Thus, similar to the specific codon bias in this region, an accumulation of polyproline motifs might allow adjustment of translational speed in order to minimize the cost of protein production.

### Polyproline motifs coordinate co-translational folding of proteins

Protein folding is a co-translational process, and it is generally believed that structural elements of a protein may influence each other during the folding process [45]. Due to the cooperativity between different parts of the structure, the timing of translation is crucial for proper folding [38]. The non-uniform distribution of synonymous codons with different translation rates fine-tunes the co-translational folding of proteins [46–54]. Fast translation of the mRNA stretches coding for structural domains helps to avoid misfolded intermediates [55], while translational pauses induced by clusters of slow-translating codons in the inter-domain linkers of multi-domain proteins facilitate independent folding of domains to minimize the chance of misfolding [46,53,56–58]. By analogy, we hypothesized that polyproline motifs may coordinate co-translational folding by slowing down translation of inter-domain linkers, and as a consequence, would be expected to occur more frequently between rather than within structural domains.

We therefore investigated the positional preference of polyproline motifs in globular multi-domain proteins. Sequence positions of 7,398 structural domains within 4,080 *E*. *coli* K-12 MG1655 proteins were obtained from Gene3D database [59]. Out of these proteins, 1,868 (45.8%) are multi-domain proteins possessing the total of 5,186 domains. An inter-domain linker was defined as the sequence span between the boundaries of two consecutive domains (if such a span was shorter than 5 amino acids, it was expanded downstream to achieve the length of 5 amino acids). This procedure yielded 3,318 inter-domain linkers between 5,186 domains.

Indeed, we found that polyproline motifs are significantly depleted in structural domains (*p*-value = 7.86e-80; fold change 0.56), but not in inter-domain linkers (*p*-value = 0.912; fold change 1.10). We then investigated the relative location of polyproline motifs with respect to domain boundaries. As seen in Fig 3B, polyproline motifs frequently occur in two regions: 1) -12 to -2 residues relative to the domain start; and 2) -2 to +9 residues relative to the domain end. Polyproline motifs are significantly enriched in these two regions (*p*-values < 0.05; fold changes for these two regions are 1.19 and 1.23, respectively). Thus, there is a strong correlation between the location of polyproline motifs and the structural domain boundaries, which was also observed for clusters of slow-translating codons [57,58,60,61]. These findings imply that the ribosome stalling effect caused by the polyproline motifs within structural domains may interfere with their folding, while stalling at domain boundaries may facilitate it.

### Polyproline motifs facilitate co-translational insertion of transmembrane helices

Another typical co-translational process is the targeting of α-helical transmembrane proteins (TPs) to the translocons, mediated by the signal recognition particle (SRP), and their insertion into the membrane [62,63]. This process has been found to be facilitated by translational pause [50,64–69]. A recent study by Fluman *et al*. identified two translation pauses, triggered by Shine-Dalgarno-like elements in *E*. *coli* mRNAs, that contribute to the SRP-mediated targeting of TPs [64]. The first pause occurs before the nascent peptide emerges from the exit tunnel of the ribosome (16 to 30 codons of the protein) and the second one occurs after the emergence of the first transmembrane helix (TMH) (-5 to +1 codons relative to the start of the second TMH). In the fungus *Emericella nidulans*, Dessen *et al*. identified two translational pauses occurring at the distance of approximately 45 and 70 codons from TMHs, caused by clusters of slow-translating codons and presumed to facilitate translocon-mediated co-translational insertion of TMH [66].

We investigated the occurrence and location of polyproline motifs in TPs. Based on the UniProt [70] annotation, we identified 912 TPs from *E*. *coli* K-12 containing the total of 5,672 TMHs. We found that 39.3% (358) of these TPs harbor polyproline motifs, which is even higher than the percentage of soluble proteins (32.6%; chi-squared test, *p*-value = 1.6e-4). No enrichment of polyproline motifs around the pause sites identified by Fluman *et al*. was observed. However, as seen in Fig 3C, we found that i) polyproline motifs rarely occur within TMH; and ii) polyproline motifs display a relatively high occurrence in four positions (positions -17 to -1, 23 to 32, 49 to 59 and 77 to 87 relative to TMH start; termed here site I, II, III and IV, respectively). The depletion of polyproline motifs in TMH is significant (*p*-value = 1.65e-27; fold change 0.39) implying that the ribosome stalling effect caused by the polyproline motifs may interfere with the folding of TPs. It should be noted that the site positions are shown relative to the start of a TMH, and thus in some cases the given region can actually be located in another TMH (see Fig 3D for illustration). We therefore tested the enrichment/depletion of the polyproline motifs in each of the four sites described above separately in TMH and in non-transmembrane regions. For example, out of the 4,439 site IV regions 3,013 and 1,426 regions are located in TMH and non-transmembrane regions, respectively. For all four sites, significant depletion of polyproline motifs was evident in TMH regions (*p*-values for sites I, II, III and IV are 2.63e-6, 1.86e-3, 7.84e-3 and 1.98e-3, respectively; fold changes of these 4 sites are 0.57, 0.56, 0.71 and 0.64, respectively), while in non-transmembrane regions a significant enrichment of polyproline motifs was observed for site III (*p*-value = 0.035; fold change 1.39). The location of this site is similar to the location of one of the translational pauses (approximately 45 codons from TMHs) identified by Dessen *et al*. Considering that most of the TMHs are 21 residues in length (S7 Fig) and that about 28 amino acids can be accommodated in the ribosome exit tunnel [71], ribosome stalling at site III may occur after the TMH has emerged from the ribosome exit tunnel and is being inserted into the membrane by translocon [63,72]. We therefore speculate that the translational pause at site III could provide a time delay for the efficient insertion of TMH.

## Discussion

Proline is a poor substrate for the ribosomal peptidyl transfer reaction [2–4], and consecutive prolines cause ribosome stalling [5]. The bacterial elongation factor P (EF-P) and its eukaryotic and archaeal orthologs e/aIF5A alleviate this stalling to some degree, but cannot fully compensate the translational burden imposed by polyproline motifs [6–8,10]. The presence of a large number of such motifs in bacterial proteomes might imply their biological significance, yet their precise functional role remains poorly understood [14,15].

In this study, we made a comprehensive attempt to shed light on the functional role of polyproline motifs by investigating their distribution and evolution in the proteomes of 43 *E*. *coli* strains. We found evidence of evolutionary selection pressure against translational stalling caused by polyproline motifs. Translational efficiency and protein abundance negatively correlate with the frequency of polyproline motifs and thus might be the driving force for their loss. Against the general trend of losing polyproline motifs during the course of evolution, we observed accumulation of polyproline motifs close to the protein N-terminus, in inter-domain regions of multi-domain proteins as well as downstream of transmembrane helices. We therefore speculate that the time gain caused by translational pause at polyproline motifs might be crucial for translational regulation, domain folding, and the proper membrane insertion, respectively.

Evolutionary selection for high efficiency of protein synthesis is one of the forces shaping mRNA sequences. For example, unequal usage of synonymous codons reflects an adaption of the codon usage to the available tRNA pool, with slow-translating codons used much more rarely than fast-translating codons [73,74]. However, protein sequence elements were also found to influence the translation rate by interacting with the ribosome exit tunnel or impairing the peptidyl transfer reaction [7,75]. An important question, which arises in this context, is whether there exists protein-level evolutionary selection for high translation efficiency. Recently, Tuller *et al*. found that short peptides, which induce ribosome stalling in yeast by interacting with the ribosomal exit tunnel, tend to be either over or underrepresented in the proteome [76]. They hypothesized that short peptide sequences were under evolutionary selection based on their synthetic efficiency. Our results show that polyproline motifs, which induce ribosome stalling by slowing down the peptidyl transfer reaction, are significantly underrepresented in *E*. *coli* proteomes, and that selection is more evident against motifs causing stronger ribosome stalling and in proteins with higher translation efficiency. These findings support the conjecture that translation efficiency-based evolutionary pressure shapes protein sequences.

Against the overall background of polyproline motif depletion, our investigation of the intra-molecular distribution pattern of polyproline motifs revealed their overrepresentation at several strategic locations, indicating their regulatory role in translation elongation. Translation elongation is a non-uniform process, which is subject to strict regulation [1,16] both in terms of the quantity of the translation products [77] and the intra-molecular variation of the elongation rate, which ensures the quality of the synthesized proteins by coordinating co-translational processes [47,64,78]. The role of polyproline motifs in the regulation of the overall translation elongation rate is exemplified by the lysine-dependent acid stress response regulator CadC of *E*. *coli* [7,16,79]. This membrane-integrated pH-sensor and transcriptional activator contains two polyproline motifs, which allow for fine-tuning of its copy number. The amount of the CadC protein is crucial for regulating the expression of the target operon. Analogously, precisely regulated translational output of the polyproline-containing receptor CpxA is required for *Shigella flexneri* virulence [80]. The intra-molecular variation of the elongation rate has so far been thought to be regulated by *cis*-acting elements embedded in the translated mRNA [57,81], such as clusters of slow-translating codons [38] and Shine-Dalgarno-like RNA sequences [64] (although the latter notion has recently been challenged [82]), as well as by *trans*-acting molecules, such as the signal recognition particle, which arrests translation elongation while targeting proteins to the membrane [83,84]. Our study highlights the role of polyproline motifs in coordinating the co-translational protein folding and transmembrane helix insertion, implying that they could serve as protein-level *cis*-acting elements, which directly regulate the rate of translation elongation.

The phenomenon we observed is not specific to *E*. *coli*. We also investigated the occurrence of polyproline motifs in *Bacillus subtilis*, a Gram-positive bacterium, and obtained qualitatively similar results (S8 Fig and S9 Fig). We therefore speculate that the polyproline motif-mediated regulation of translation elongation may be universal in bacteria. *B*. *subtilis* has a lower occurrence of polyproline motifs than *E*. *coli* (average fold change 0.73 vs 0.82), which may indicate a stronger evolutionary selection against polyproline motifs. We initiated a follow-up study of polyproline motif occurrence in bacteria and also in eukaryotes, although the ribosome arresting sequences in eukaryotes are not limited to consecutive prolines [85,86]. In another follow up study we are investigating the interplay between the RNA level elements, such as codon usage and RNA structure, and polyproline motifs (Qi, F. *et al*., in preparation).

## Materials and methods

### Proteomes and orthologous groups of *E*. *coli*

We obtained *Escherichia coli* proteomes and orthology assignments from the OMA database [87]. The total of 206,360 protein sequences from six out of seven *E*. *coli* phylotypes [88] were downloaded (S2 Table). We also obtained 11,356 orthologous groups covering 195,056 proteins.

### The core- and accessory proteomes of *E*. *coli*

The core- and accessory proteomes were defined based on the occurrence of orthologous groups. An orthologous group was classified as belonging to the core proteome if it was present in all the 43 *E*. *coli* proteomes, otherwise it was considered to belong to the accessory proteome. All proteins not assigned to any orthologous group were classified as belonging to the accessory proteome. This procedure yielded a core proteome of *E*. *coli* covering 73,745 proteins and an accessory proteome covering 132,615 proteins.

### Identification of polyproline motifs in real and random sequences

Using the program *fuzzpro* from the EMBOSS package [89] we identified polyproline motifs in the *E*. *coli* proteins. The same procedure was applied to randomly generated sequences. Each amino acid sequence in our dataset was shuffled 1,000 times while maintaining its composition using the program *shuffleseq* from the EMBOSS package [89], yielding 1,000 sets of random *E*. *coli* protein sequences.

### Enrichment and depletion of polyproline motifs

We used the SPatt algorithm [90] to assess the enrichment and depletion of polyproline motifs, taking into account occurrence patterns of proline in various parts of protein structure. SPatt determines the expected occurrence of a sequence motif based on a Markov chain model of order *m* (model M*m*), compares the observed occurrence with expected one, and calculates the *p*-value for the significance of a motif’s enrichment or depletion. Choosing a model M*m* means taking into account the *m*-mer and (*m*+1)-mer compositions while determining the expected occurrence. For example, the model M0 solely takes into account the amino acid composition, while choosing the model M1 takes into account the compositions of amino acid monomers and dimers. For a motif of length *l*, the maximum *m* is (*l*-2). In our case, although a polyproline motif can have more than 2 residues, the essential part of a polyproline motif is the proline stretch with at least two consecutive proline residues. Therefore, we chose model M0 in our tests.

### Normalization of polyproline motif occurrence

The occurrence of polyproline motifs in proteins was normalized by the polyproline motif occurrence in randomly generated sequences. Each amino acid sequence (either full protein sequences or specific sequence segments of interest) was shuffled 1,000 times while maintaining its composition using the program *shuffleseq* from the EMBOSS package [89], yielding 1,000 sets of random sequences. The number of times the polyproline motif occurred in a real sequence was then divided by the number of times the same motif occurred in each of the 1,000 random sequences, yielding a vector of 1,000 ratios between the observed and the expected polyproline motif occurrence. The Mann-Whitney-Wilcoxon test was employed to assess the significance of the difference between two such vectors corresponding to two different sequences or sequence segments. This procedure was carried out for each strain of *E*. *coli* separately.

### Fold change of polyproline motif occurrence

The fold change of polyproline motif occurrence is used as a measure of the enrichment/depletion level of polyproline motifs. It is defined as the ratio between the observed and expected occurrence of polyproline motifs, and is calculated as:

$$Fold\_change=\frac{N_{obs}}{N_{exp}}$$ (1)

Where *N*_*obs*_ and *N*_*exp*_ are the observed and expected occurrences of polyproline motifs, respectively. The *N*_*exp*_ is either the mean value of the polyproline motif occurrences in 1,000 sets of random sequences (for fold change of whole proteomes; see *Normalization of polyproline motif occurrence* for detail) or the mean of the distribution of expected motif occurrence calculated by SPatt algorithm (for fold change of structural domains, domain linkers, TMHs and regions bracketing domains and TMHs; see *Enrichment and depletion of polyproline motifs* for detail).

### Classification of polyproline motifs

Polyproline motifs were classified into three groups (strong, medium and week) according to their predicted ribosomal translation arrest strength. The prediction is based on experimental data both from systematic *in vitro* and *in vivo* analyses [10,12,14–16] (S3 Table and S4 Table).

As described in the *Introduction* section, the ribosome stalling strength of a X_(-2)_X_(-1)_-nP-X_(+1)_ motif is dependent on the number of consecutive prolines and on the flanking amino acids. First, we classified the flanking residues X_(-2)_, X_(-1)_ and X_(+1)_ (motifs involving ambiguous amino acids were excluded from consideration) according to their influence on the ribosome stalling strength. If a flanking residue of the polyproline motif in an *E*. *coli* strain lacking *efp* (Δ*efp*) was responsible for a decrease of the translational output by ≥70% compared to a wildtype control, the residue was defined as strong [5,10,12]. In cases where the protein synthesis was reduced by 30–60%, we classified the stalling strength as medium. In all other cases, the polyproline sequence context was assumed to cause only a weak arrest. All possible X_(-2)_X_(-1)_-nP-X_(+1)_ motifs and their respective arrest strength are listed in S4 Table and S5 Table. Based on our classification, we correlated the predicted motif strength to available ribosome profiling data [10]. Woolstenhulme *et al*. compared the ribosome occupancy at a diprolyl motif with the occupancy downstream of the motif in an Δ*efp* strain [10]. Stalling was ranked according to the observed assymetry (ratio) between these two values. When setting an assymetry quotient of 2.00 as a threshold for proteins subject to strong translation arrest, we found that more than 75% of them possess at least one medium or strong polyproline motif. This number further increases to ~80% and ~90% when applying more stringent cutoffs of 3.00 and 5.00 to the assymetry score, respectively (S6 Table).

### Word frequency in protein sequences

Frequencies of each single and dimer amino acid in protein sequences were calculated using the *compseq* program from the EMBOSS package [89]. For each amino acid dimer, an expected frequency was additionally calculated based on the observed frequencies of single amino acids.

### Multiple alignment of protein sequences

Multiple alignments of protein sequences in each orthologous group were computed using the Clustal Omega software [91] with all default parameters.

### Construction of phylogenetic trees

Phylogenetic trees for each orthologous group with at least three proteins containing at least one polyproline motif were reconstructed using the PhyML software [92]. These trees were then rooted at midpoint.

### Reconstruction of evolutionary events

In order to reconstruct the gain and loss of the ribosome stalling effect in the evolutionary history of *E*. *coli* protein families, we first assigned one of the four possible ribosome stalling states [S (strong), M (medium), W (weak) and N (none)] to all the exterior nodes (leaves) of the phylogenetic trees. Subsequently, the Maximum Likelihood algorithm [93] was employed to reconstruct the states of ancestral nodes (internal nodes). The change of state between a given node and its ancestral node from a stronger stalling effect state to a weaker or no stalling effect state was defined as a loss of the stalling effect, while the change from a weaker or no stalling effect state to a stronger state was defined as a gain event.

### Propensity of stalling effect change

We defined propensity of stalling effect change (PSEC) similar to propensity of gene loss (PGL) frequently used in evolutionary studies [94]. PGL captures the idea that the longer the time during which a gene could have been lost but was not, the lower the propensity of this gene to be lost. PGL is thus defined as the ratio between the total length of branches in which the gene is lost and the total length of branches in which the gene could have been lost [95,96]. Similarly, PSEC captures the idea that the longer the time the stalling effect of a motif could have been gained/lost but was not, the lower the propensity of the stalling effect to be gained/lost. However, our model is somewhat more complex than the PGL model, since the PGL only considers gene loss and we have to consider both gain and loss of the stalling effect. Therefore, the PSEC is calculated as the difference between the propensities of gain and loss of the stalling effect:

$$PSEC=\frac{\sum B_{g}}{\sum B_{cg}}-\frac{\sum B_{l}}{\sum B_{cl}}$$ (2)

where *B*_*g*_ and *B*_*l*_ are the lengths of the branches in which the stalling effect was gained and lost, respectively, and *B*_*cg*_ and *B*_*cl*_ are the lengths of branches in which the stalling effect could have been gained and lost, respectively. Thus, a positive PSEC indicates that the stalling effect of a sequence motif tends to be gained, while a negative PSEC indicates that it tends to be lost during evolution.

### Protein abundance, gene expression, and translation efficiency

Protein abundance data used in this study was from [97,98], covering 2,163 proteins. Microarray data on transcription levels of 2,710 genes from *E*. *coli* K-12 MG1655 under standard growth conditions was downloaded from the ASAP database [99]. Translation efficiency for each of the 1,743 genes present in both datasets was calculated as:

$${Translation\_efficiency}_{i}=\frac{{Protein\_abundance}_{i}}{{Transcription\_level}_{i}}$$ (3)

### Domain composition of the *E*. *coli* proteins

Sequence positions of 7,398 structural domains in 4,080 *E*. *coli* K-12 MG1655 proteins were obtained from the Gene3D database [59].

### Transmembrane segments

We obtained the sequence positions of 5,672 transmembrane segments within 912 α-helical transmembrane proteins from the UniProt database [70]. Since reviewed data on transmembrane proteins of *E*. *coli* K-12 MG1655 (taxonomy ID 511145) are not available in the UniProt database, we used the reviewed data of *E*. *coli* K-12 (taxonomy ID 83333) instead.

## Supporting information

S1 Fig Numbers of *E*. *coli* proteins with 1, 2 and >2 polyproline motifs. (PDF)

S2 Fig Occurrence of polyproline motifs is lower than the random level. The histogram shows the numbers of motifs found in 1,000 sets of random sequences and the blue line shows the number of motifs found in real sequences. The results for 42 *E*. *coli* strains (except for *E*. *coli* K-12 MG1655) are shown. The OMA id and fold change for each strain are shown in the panel title. For mapping OMA ids to names of strains, please see S2 Table.
(PDF)

S3 Fig Numbers of polyproline motifs negatively correlate with the strength of the ribosome stalling effect. The results for 42 *E*. *coli* strains (except for *E*. *coli* K-12 MG1655) are shown. The OMA id of each strain is shown in the panel title. For mapping OMA ids to names of strains, please see S2 Table. All the differences are significant according to Mann-Whitney-Wilcoxon test, *p*-values < 2.2e-16.
(PDF)

S4 Fig Occurrence of polyproline motifs in the core proteome is lower than that in the accessory proteome. The results for 42 *E*. *coli* strains (except for *E*. *coli* K-12 MG1655) are shown. The OMA id of each strain is shown in the panel title. For mapping OMA ids to names of strains, please see S2 Table. All the differences are significant according to Mann-Whitney-Wilcoxon test, *p*-values < 2.2e-16.
(PDF)

S5 Fig Occurrences of polyproline motifs in the core/accessory proteome in the 43 *E*. *coli* strains. The OMA id of each strain is shown on the x-axis. For mapping OMA ids to the names of strains, please see S2 Table. Fold changes for the core proteome: mean 0.76, standard deviation 0.003. Fold changes for the accessory proteome: mean 0.85. standard deviation 0.023.
(PDF)

S6 Fig Occurrence of polyproline motifs is correlated with the number of proteins in the proteomes. Pearson’s r = 0.68, *p*-value = 4.82e-7.
(PDF)

S7 Fig Histogram of the TMH length. (PDF)

S8 Fig Occurrence of polyproline motifs in *B*. *subtilis* is lower than the random level. The histogram shows the numbers of motifs found in 1,000 sets of random sequences and the blue line shows the number of motifs found in real sequences. The name and fold change of each strain are shown in the panel title. The proteomes of the 4 *B*. *subtilis* strains including 16,678 proteins were downloaded from the OMA database [87].
(PDF)

S9 Fig The distribution of polyproline motifs in *B*. *subtilis* is qualitatively similar to *E*. *coli*. The strain 168 were used as a reference strain of *B*. *subtilis*. (A) Occurrence of polyproline motifs in the first 50 residues is higher than elsewhere in the protein sequence (Mann-Whitney-Wilcoxon test, *p*-value < 2.2e-16; fold change 0.82 vs 0.73). Error bars indicate the standard deviation. (B) Occurrence of polyproline motifs is associated with domain boundaries. Regions with relatively high motif occurrence are marked red. Data are smoothed over a three-residue window. Left: frequency of motifs relative to domain start (dashed line). Right: frequency of motifs relative to domain end (dashed line). Sequence positions of 6,086 structural domains in 3,076 *B*. *subtilis* strain 168 proteins were obtained from the Gene3D database [59]. (C) Frequency of polyproline motifs relative to the start position of TMH. TMH is marked green (assuming the typical length of 21 residues). Regions with high motif frequency are marked red. Data are smoothed over a three-residue window. The sequence positions of 5,456 transmembrane segments within 984 α-helical transmembrane proteins of *B*. *subtilis* strain 168 were obtained from the UniProt database [70]. (D) The typical length of TMHs in *B*. *subtilis* proteins is 21 residues.
(PDF)

S1 Table The list of 2,115 polyproline motifs identified in *E*. *coli* K-12 MG1655. (XLSX)

S2 Table List of all 43 *E*. *coli* strains used in this study. (XLSX)

S3 Table Classification of the effect amino acids at position X_(-2),_ X_(-1)_ and X_(+1)_ exert on ribosomal stalling strength. (PDF)

S4 Table Rules for the prediction of ribosomal stalling strength induced by a X_(-2)_X_(-1)_-nP-X_(+1)_ motif. (PDF)

S5 Table Motif classification. (XLSX)

S6 Table Matching the predicted stalling strength of polyproline motifs with the ribosome profiling data from C. J. Woolstenhulme [Cell Rep 11:13–21, 2015]. (XLSX)
