# Supplementary figures and images for: Evolutionary analysis of polyproline motifs in Escherichia coli reveals their regulatory role in translation
Source: PLoS Comput Biol. 2018 Feb 1;14(2):e1005987. doi: 10.1371/journal.pcbi.1005987 (PMC5811046; doi:10.1371/journal.pcbi.1005987)

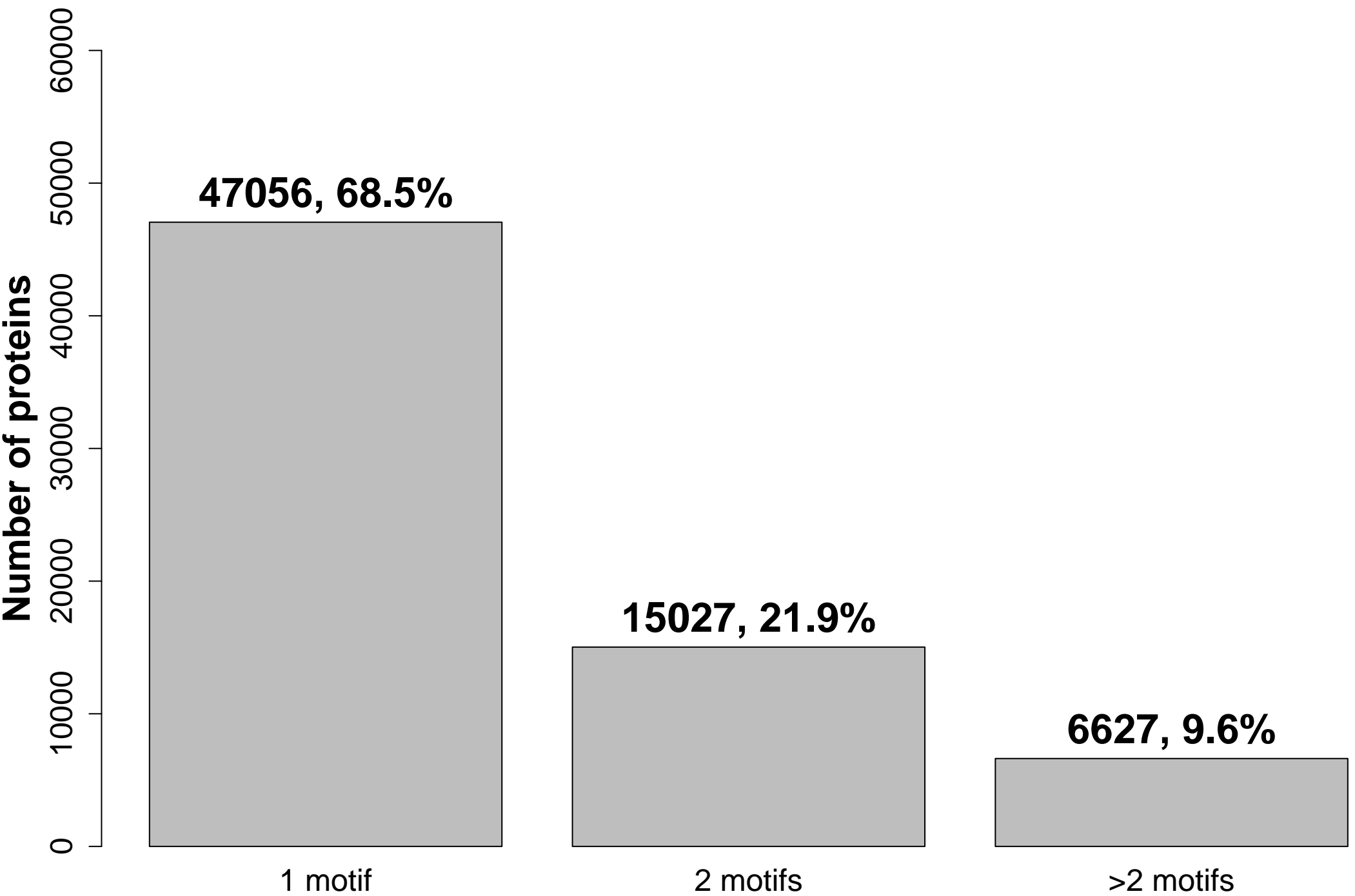

Supplement: S1 Fig — (PDF) [file pcbi.1005987.s001.pdf]

Number of random sequence sets

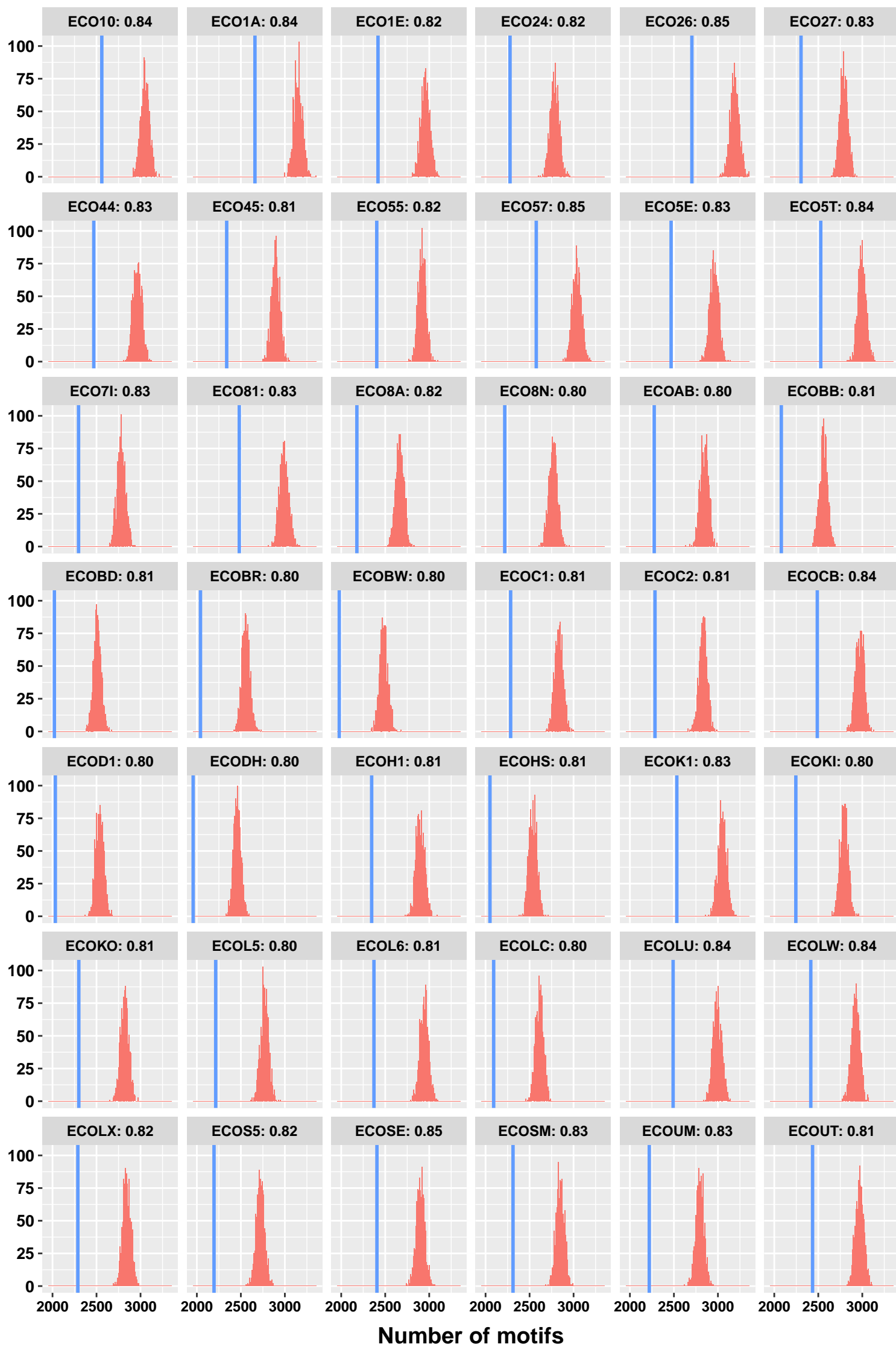

Supplement: S2 Fig — The histogram shows the numbers of motifs found in 1,000 sets of random sequences and the blue line shows the number of motifs found in real sequences. The results for 42 E. coli strains (except for E. coli K-12 MG1655) are shown. The OMA id and fold change for each strain are shown in the panel title. For mapping OMA ids to names of strains, please see S2 Table. (PDF) [file pcbi.1005987.s002.pdf]

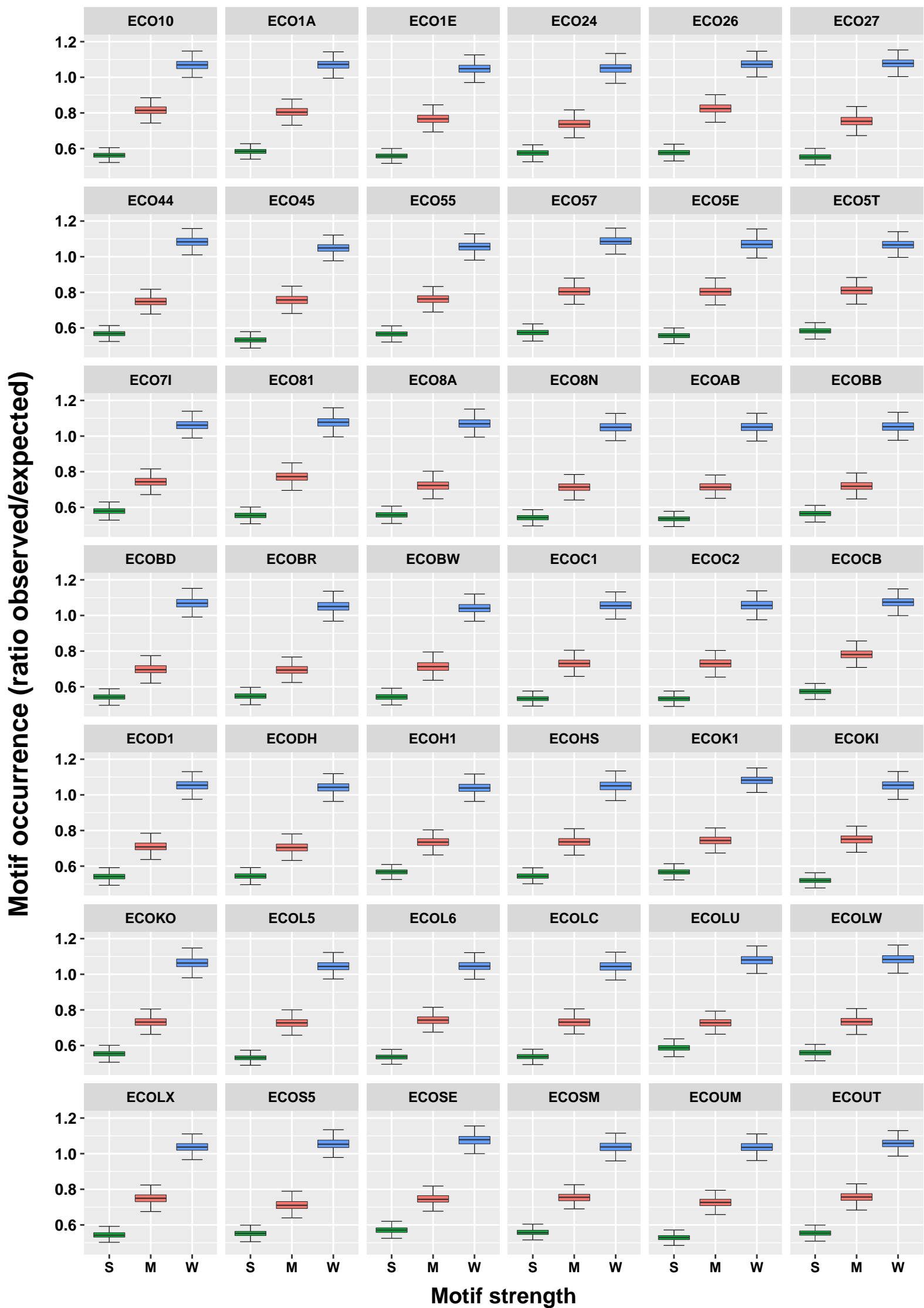

Supplement: S3 Fig — The results for 42 E. coli strains (except for E. coli K-12 MG1655) are shown. The OMA id of each strain is shown in the panel title. For mapping OMA ids to names of strains, please see S2 Table. All the differences are significant according to Mann-Whitney-Wilcoxon test, p-values < 2.2e-16. (PDF) [file pcbi.1005987.s003.pdf]

Motif occurrence (ratio observed/expected)

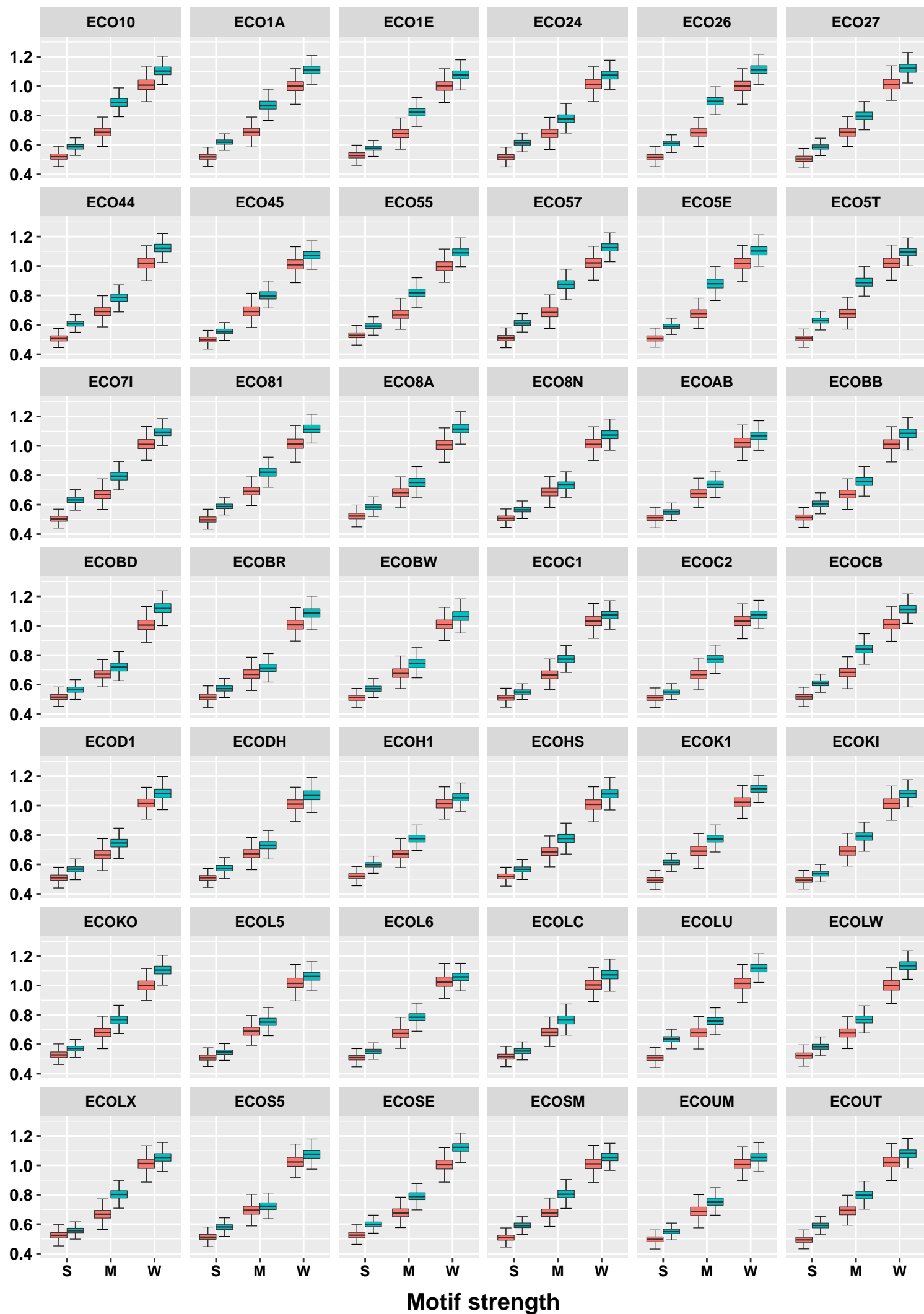

Proteome ■ core ■ accessory

Supplement: S4 Fig — The results for 42 E. coli strains (except for E. coli K-12 MG1655) are shown. The OMA id of each strain is shown in the panel title. For mapping OMA ids to names of strains, please see S2 Table. All the differences are significant according to Mann-Whitney-Wilcoxon test, p-values < 2.2e-16. (PDF) [file pcbi.1005987.s004.pdf]

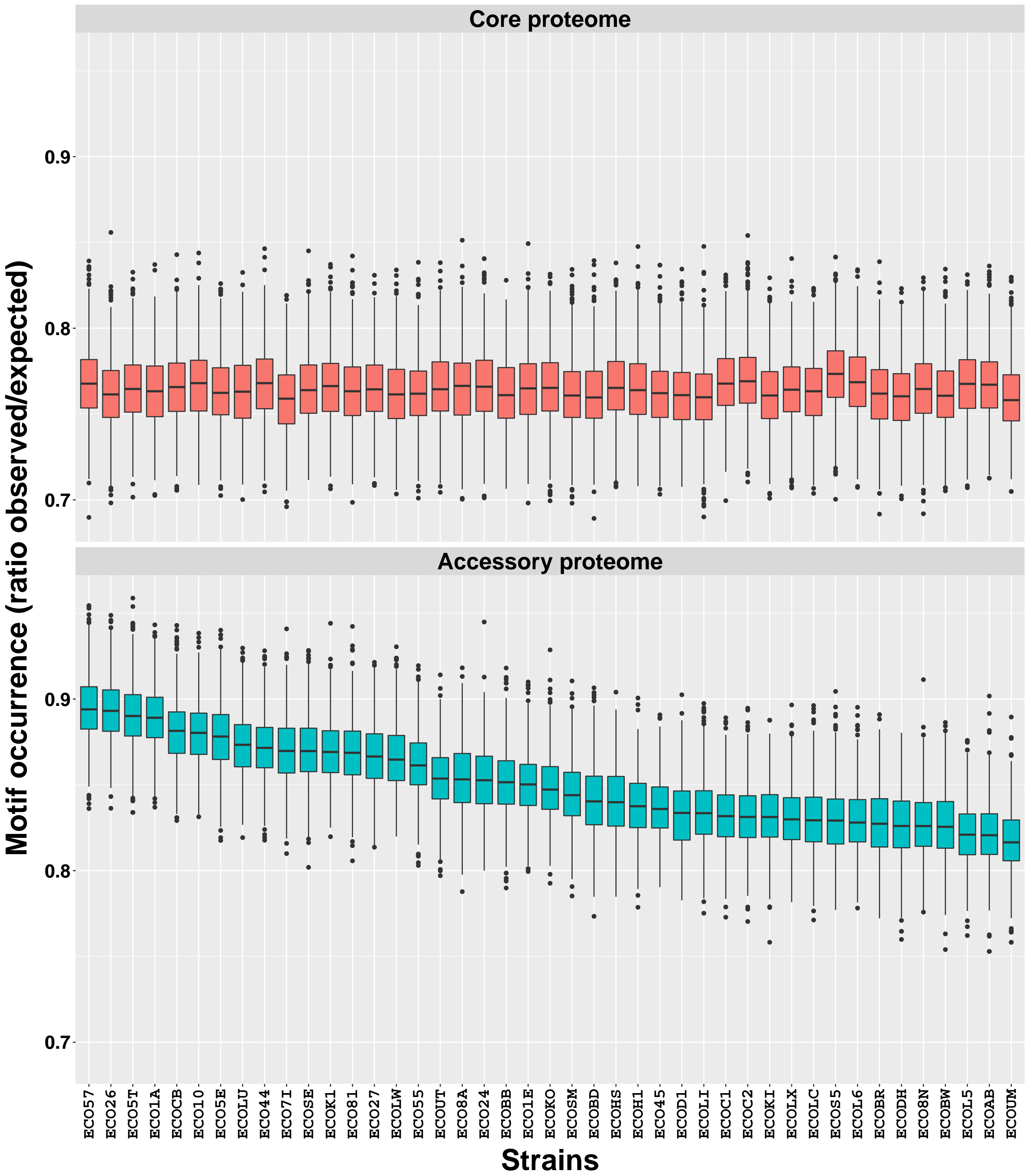

Supplement: S5 Fig — The OMA id of each strain is shown on the x-axis. For mapping OMA ids to the names of strains, please see S2 Table. Fold changes for the core proteome: mean 0.76, standard deviation 0.003. Fold changes for the accessory proteome: mean 0.85. standard deviation 0.023. (PDF) [file pcbi.1005987.s005.pdf]

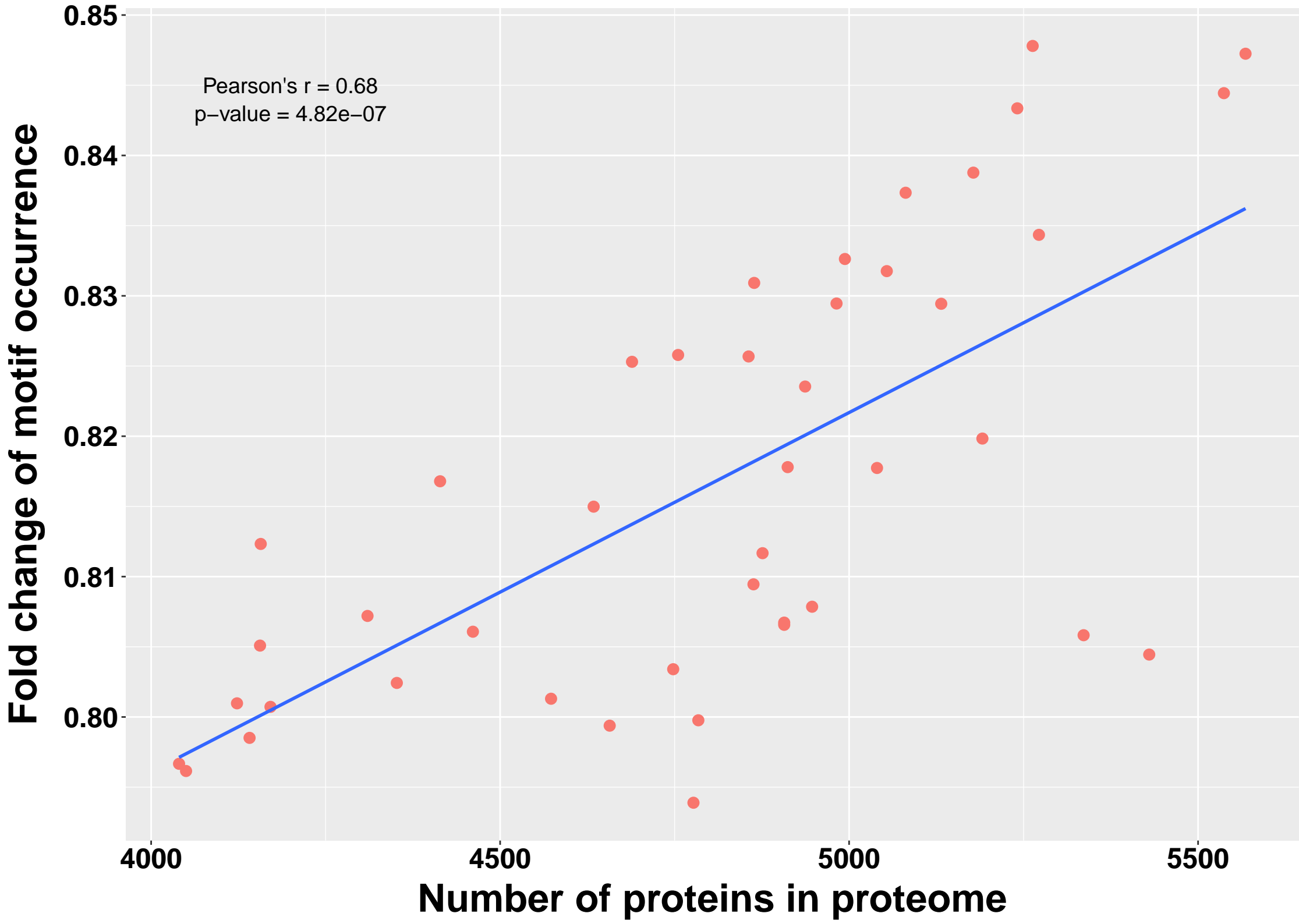

Supplement: S6 Fig — Pearson’s r = 0.68, p-value = 4.82e-7. (PDF) [file pcbi.1005987.s006.pdf]

**Number of TMH**

4000  
3000  
2000  
1000  
0

10

20

30

**Length of TMH**

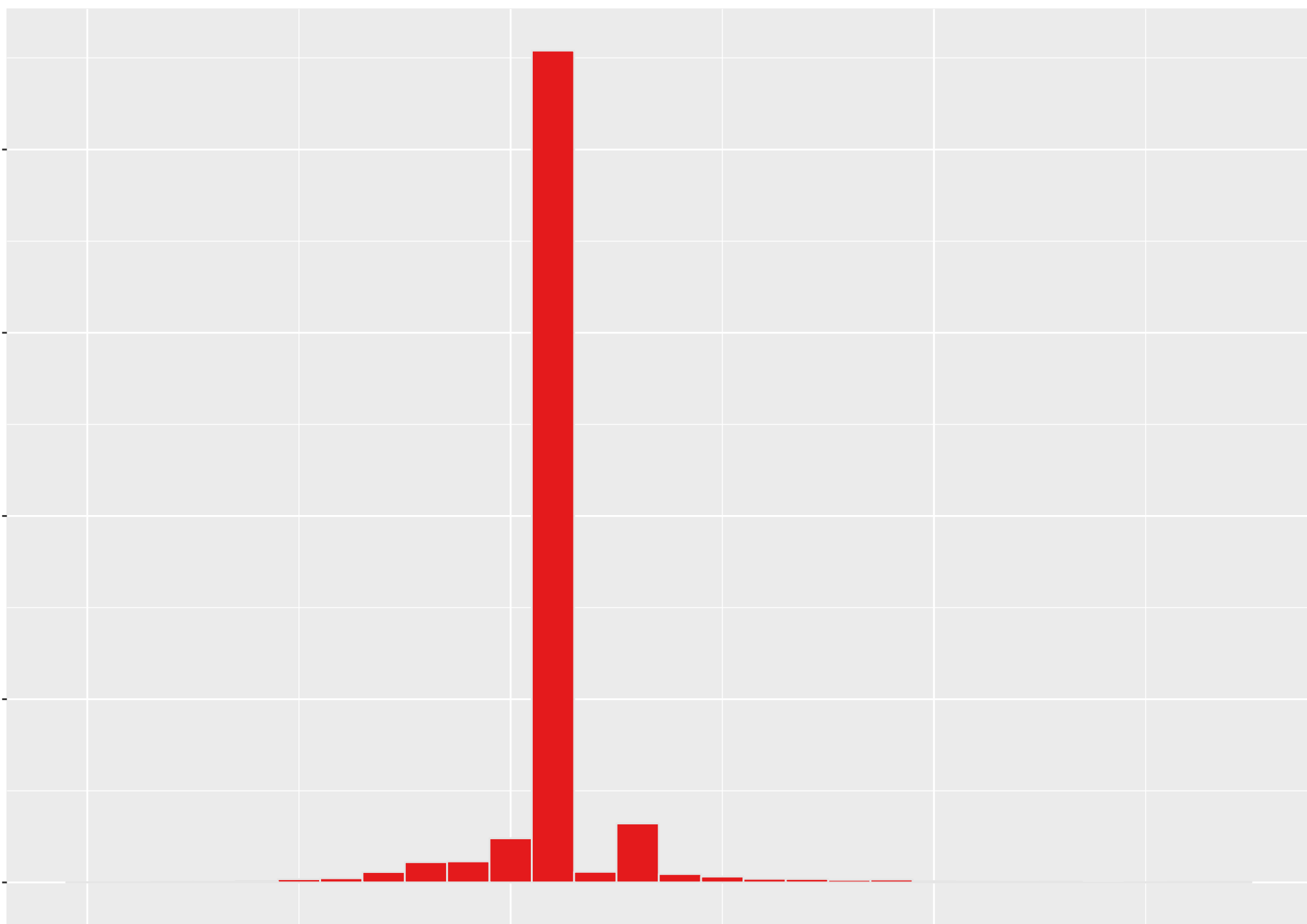

Supplement: S7 Fig — (PDF) [file pcbi.1005987.s007.pdf]

Number of random sequence sets

strain 168: 0.74

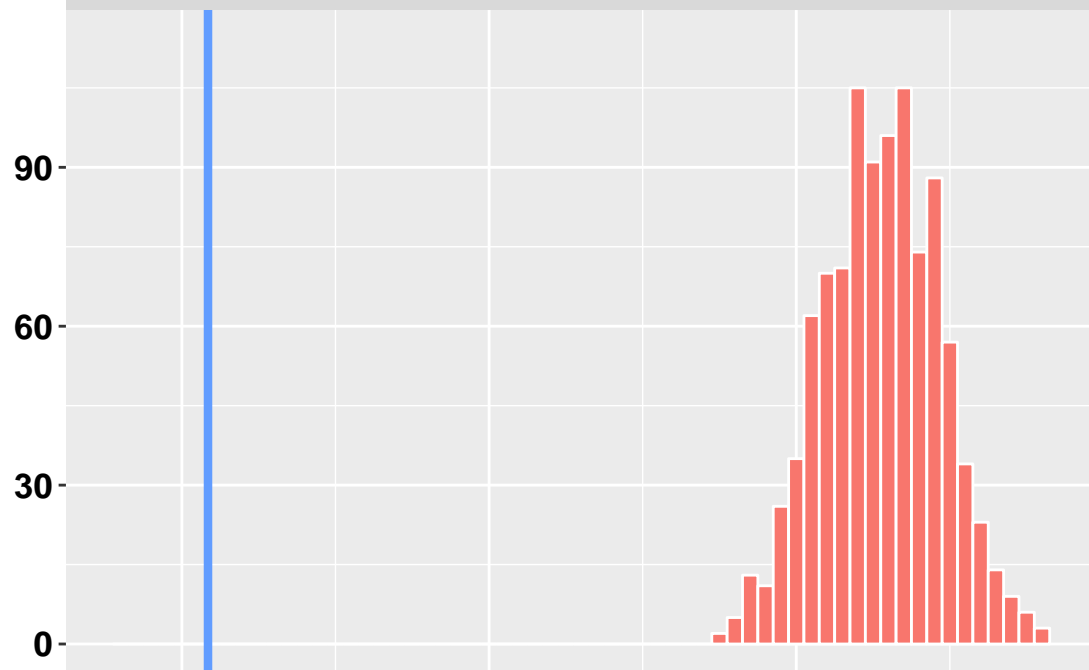

strain BSn5: 0.73

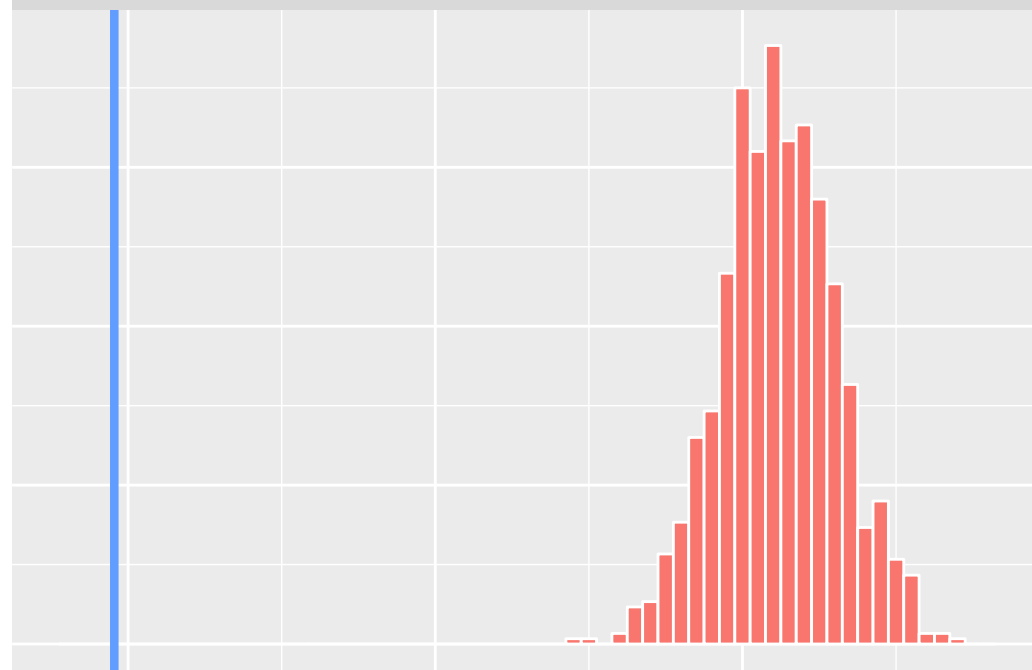

strain TU-B-10: 0.73

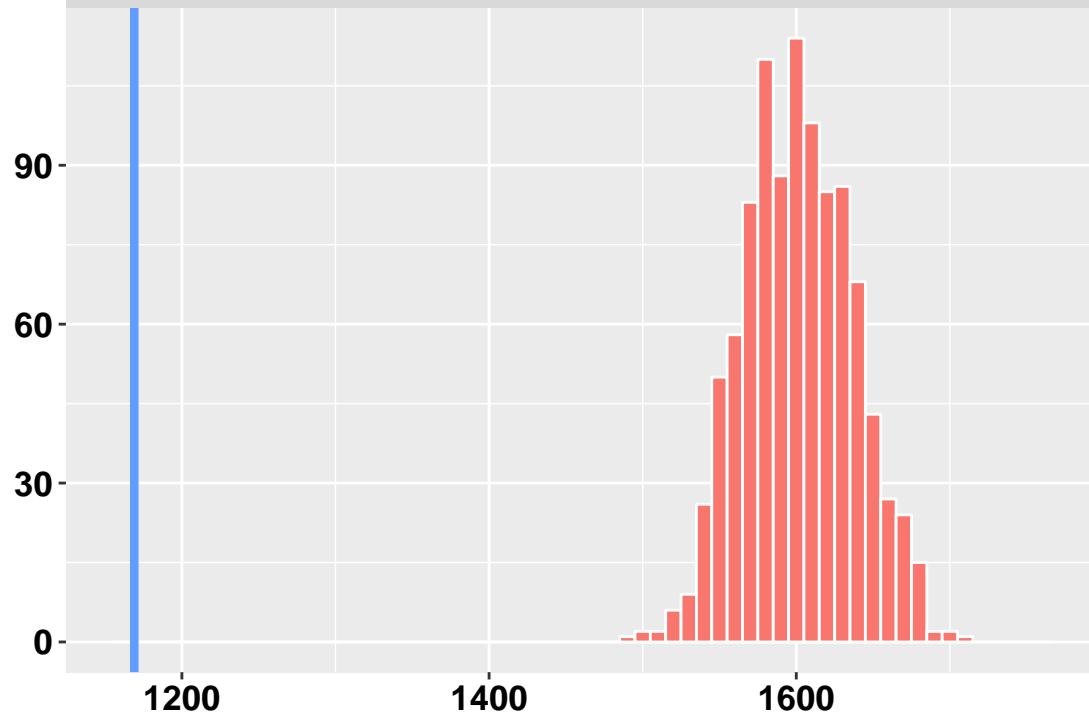

strain W23: 0.72

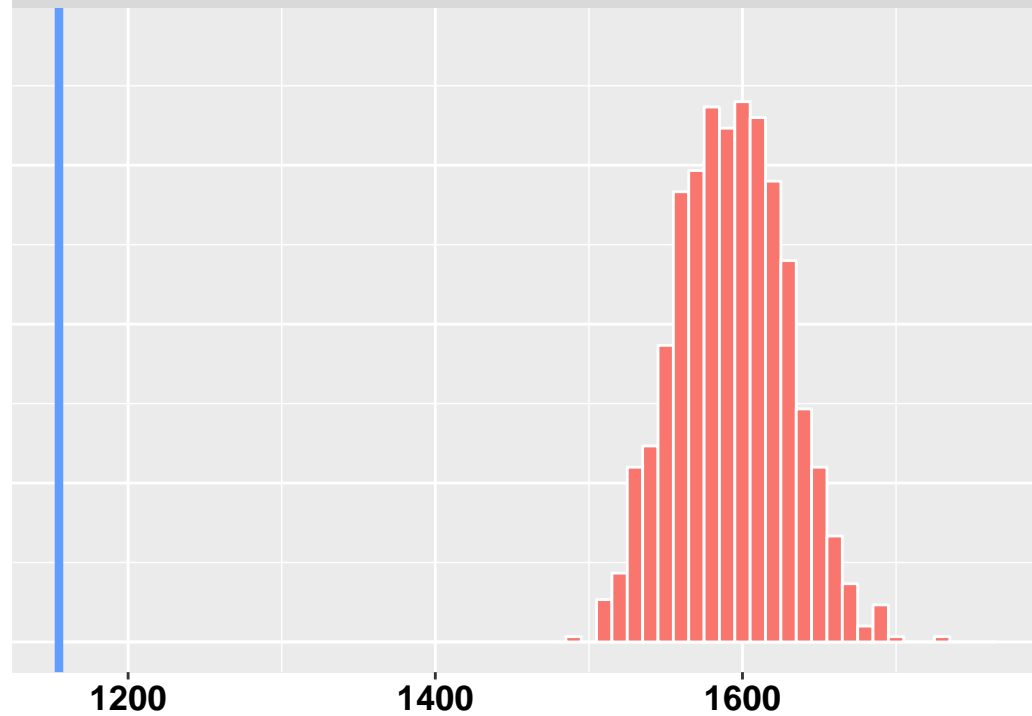

Number of motifs

Supplement: S8 Fig — The histogram shows the numbers of motifs found in 1,000 sets of random sequences and the blue line shows the number of motifs found in real sequences. The name and fold change of each strain are shown in the panel title. The proteomes of the 4 B. subtilis strains including 16,678 proteins were downloaded from the OMA database [87]. (PDF) [file pcbi.1005987.s008.pdf]

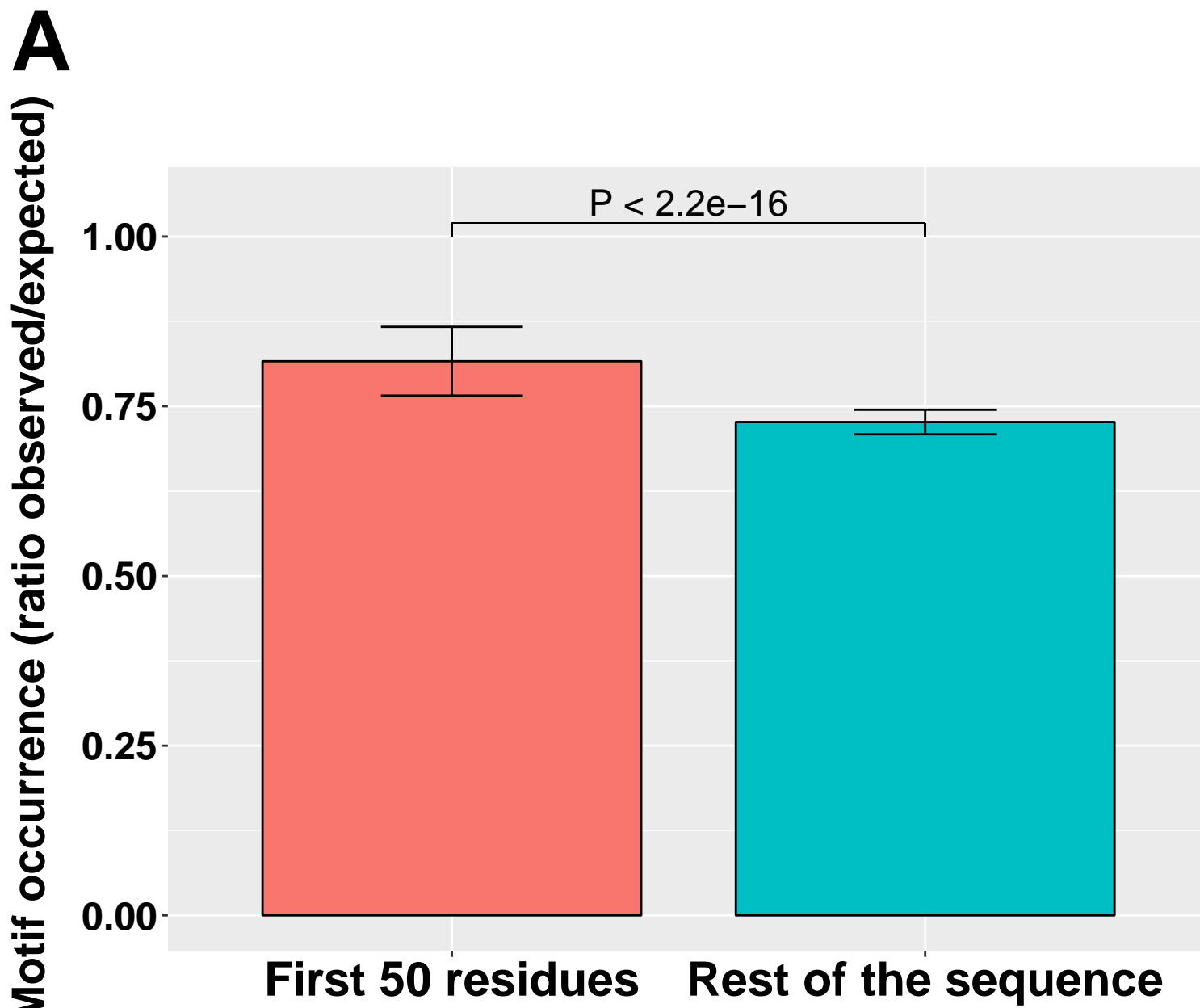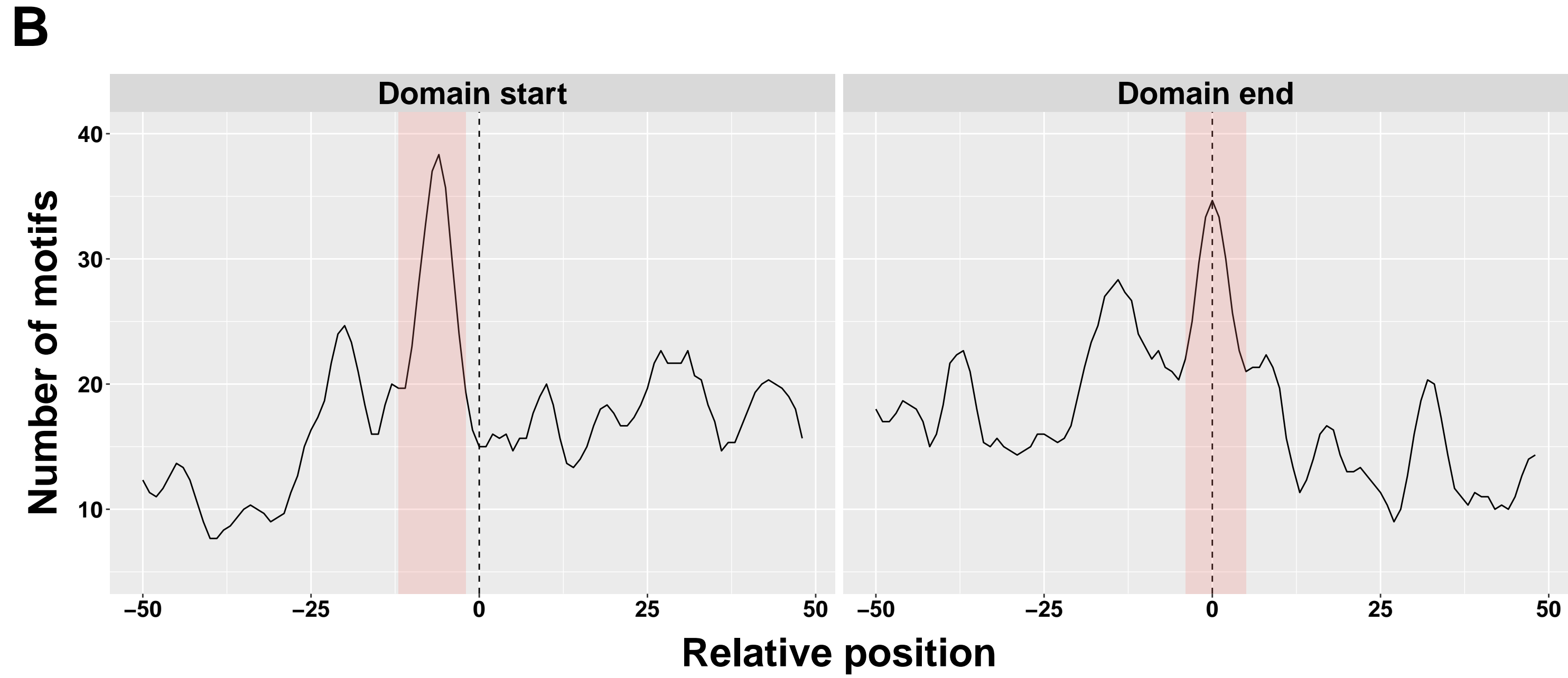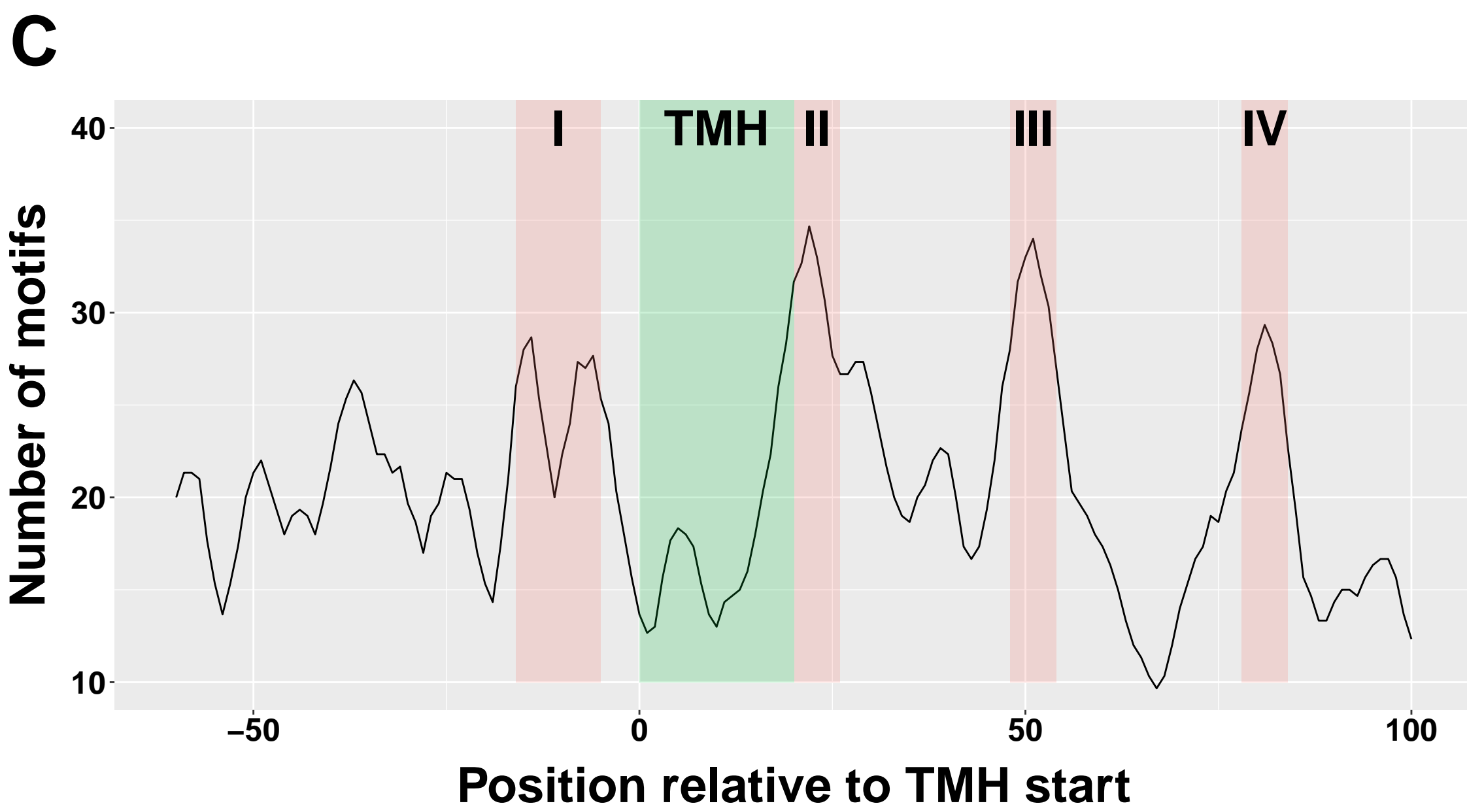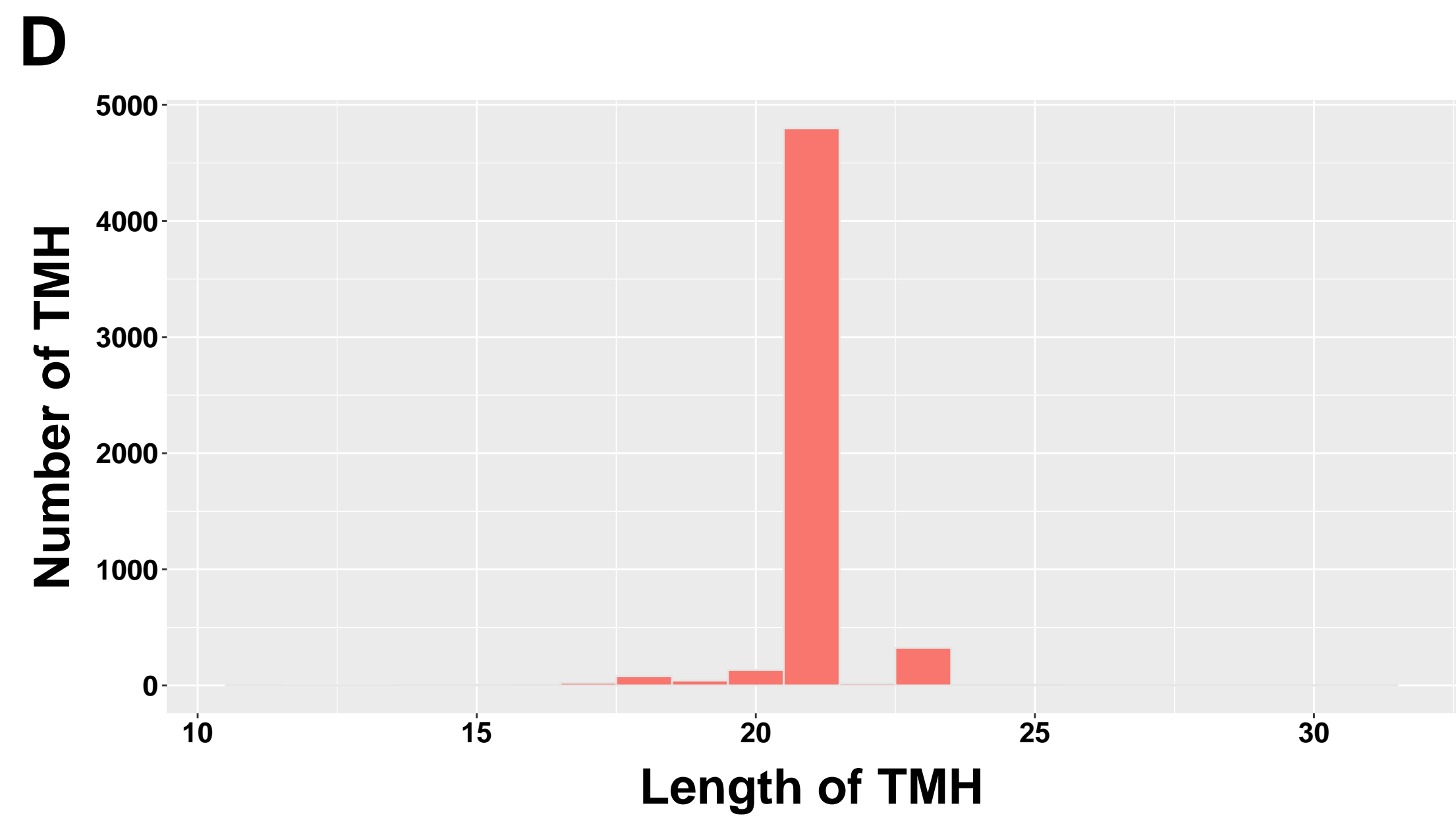

Supplement: S9 Fig — The strain 168 were used as a reference strain of B. subtilis. (A) Occurrence of polyproline motifs in the first 50 residues is higher than elsewhere in the protein sequence (Mann-Whitney-Wilcoxon test, p-value < 2.2e-16; fold change 0.82 vs 0.73). Error bars indicate the standard deviation. (B) Occurrence of polyproline motifs is associated with domain boundaries. Regions with relatively high motif occurrence are marked red. Data are smoothed over a three-residue window. Left: frequency of motifs relative to domain start (dashed line). Right: frequency of motifs relative to domain end (dashed line). Sequence positions of 6,086 structural domains in 3,076 B. subtilis strain 168 proteins were obtained from the Gene3D database [59]. (C) Frequency of polyproline motifs relative to the start position of TMH. TMH is marked green (assuming the typical length of 21 residues). Regions with high motif frequency are marked red. Data are smoothed over a three-residue window. The sequence positions of 5,456 transmembrane segments within 984 α-helical transmembrane proteins of B. subtilis strain 168 were obtained from the UniProt database [70]. (D) The typical length of TMHs in B. subtilis proteins is 21 residues. (PDF) [file pcbi.1005987.s009.pdf]
