# Supplementary material for: Evolutionary analysis of polyproline motifs in Escherichia coli reveals their regulatory role in translation
Source: PLoS Comput Biol. 2018 Feb 1;14(2):e1005987. doi: 10.1371/journal.pcbi.1005987 (PMC5811046; doi:10.1371/journal.pcbi.1005987)
Supplement: S3 Table — (PDF) [file pcbi.1005987.s012.pdf]

S3 Table. Classification of the effect amino acids at position $X_{(-2)}$, $X_{(-1)}$ and $X_{(+1)}$ exert on ribosomal stalling strength.

Reference: [1]

Effect on stalling strength	Amino acid at position $X_{(-2)}$	Amino acid at position $X_{(-1)}$		Amino acid at position $X_{(+1)}$
Strong	A, E, H, I, K, P, R, V	D, G, P	**PP**	D, E, K, N, P, S, W
Moderate	D, G, N, Q, Y	A	**PP**	A, G, Q,
Weak	C, F, L, M, T, S	C, E, F, H, I, K, L, M, N, Q, R, S, T, V, W, Y	**PP**	C, F, H, I, L, M, R, T, V, Y

Reference: [2,3]

Effect on stalling strength	Amino acid at position $X_{(-2)}$	Amino acid at position $X_{(-1)}$		Amino acid at position $X_{(+1)}$
Strong	D, E, H, K, P, Q, R, Y, W	A, D, P	**PP**	D, G, N, P, W
Moderate	F, G, I, M, N, V	E, G, S	**PP**	E, Q, S, T
Weak	A, C, L, S, T	C, F, H, I, K, L, M, N, Q, R, T, V, W, Y	**PP**	A, C, F, H, I, K, L, M, R, V, Y

Combined data

Effect on stalling strength	Amino acid at position $X_{(-2)}$	Amino acid at position $X_{(-1)}$		Amino acid at position $X_{(+1)}$
strong	A, D, E, G, H, I, K, P Q, R, V, W, Y	A, D, G, (P)	**nP**	D, E, G, N, (P), S, W
Moderate	F, M, N	S, E	**nP**	A, K, Q, T
Weak	C, L, S, T	C, F, H, I, K, L, M, N, Q, R, T, V, W, Y	**nP**	C, F, H, I, L, M, R, V, Y

Reference

1. Woolstenhulme CJ, Guydosh NR, Green R, Buskirk AR. High-precision analysis of translational pausing by ribosome profiling in bacteria lacking EFP. Cell Rep. 2015;11: 13–21. doi:10.1016/j.celrep.2015.03.014

2. Peil L, Starosta AL, Lassak J, Atkinson GC, Virumäe K, Spitzer M, et al. Distinct XPPX sequence motifs induce ribosome stalling, which is rescued by the translation elongation factor EF-P. Proc Natl Acad Sci U S A. 2013;110: 15265–70. doi:10.1073/pnas.1310642110
3. Starosta AL, Lassak J, Peil L, Atkinson GC, Virumäe K, Tenson T, et al. Translational stalling at polyproline stretches is modulated by the sequence context upstream of the stall site. Nucleic Acids Res. 2014;42: 10711–9. doi:10.1093/nar/gku768
