# Supplementary material for: Evolutionary analysis of polyproline motifs in Escherichia coli reveals their regulatory role in translation
Source: PLoS Comput Biol. 2018 Feb 1;14(2):e1005987. doi: 10.1371/journal.pcbi.1005987 (PMC5811046; doi:10.1371/journal.pcbi.1005987)
Supplement: S4 Table — (PDF) [file pcbi.1005987.s013.pdf]

S4 Table. Rules for the prediction of ribosomal stalling strength induced by a $X_{(-2)}X_{(-1)}$-nP-$X_{(+1)}$ motif.

I. Rules for n > 3: all motifs are classified as strong

II. Rules for n = 3: put the first proline at $X_{(-1)}$ position					
**$X_{(-2)}$**	**$X_{(-1)}$**		**$X_{(+1)}$**	**motif strength**	**Example**
strong	**P**	**PP**	strong	strong	KPPPD
strong	**P**	**PP**	medium	strong	KPPPK
strong	**P**	**PP**	weak	strong	KPPPC
**$X_{(-2)}$**	**$X_{(-1)}$**		**$X_{(+1)}$**	**motif strength**	**Example**
medium	**P**	**PP**	strong	strong	FPPPD
medium	**P**	**PP**	medium	medium	FPPPK
medium	**P**	**PP**	weak	medium	FPPPC
**$X_{(-2)}$**	**$X_{(-1)}$**		**$X_{(+1)}$**	**motif strength**	**Example**
weak	**P**	**PP**	strong	strong	SPPPD
weak	**P**	**PP**	medium	medium	SPPPK
weak	**P**	**PP**	weak	weak	SPPPC

III. Rules for n = 2					
**If $X_{(+1)}$ = strong, classify $X_{(-1)}$ using the rule for $X_{(-2)}$**			**$X_{(+1)}$**	**motif strength**	**Example**
strong		**PP**	strong	strong	KPPD
medium		**PP**	strong	medium	FPPD
weak		**PP**	strong	weak	SPPD
**$X_{(-2)}$**	**$X_{(-1)}$**		**$X_{(+1)}$**	**motif strength**	**Example**
strong	strong	**PP**	medium	strong	KGPPK
strong	medium	**PP**	medium	medium	KEPPK
medium	strong	**PP**	medium	medium	FGPPK
medium	medium	**PP**	medium	medium	FEPPK
**If $X_{(+1)}$ = medium and $X_{(-2)}$ or $X_{(-1)}$ = weak, classify $X_{(-1)}$ using the rule for $X_{(-2)}$**			**$X_{(+1)}$**	**motif strength**	**Example**
strong		**PP**	medium	medium	TIPPK
medium		**PP**	medium	medium	AFPPK
weak		**PP**	medium	weak	ACPPK
**$X_{(-2)}$**	**$X_{(-1)}$**		**$X_{(+1)}$**	**motif strength**	**Example**
strong	strong	**PP**	weak	strong	KGPPC
strong	medium	**PP**	weak	medium	KEPPC
strong	weak	**PP**	weak	weak	KCPPC
medium	strong	**PP**	weak	medium	FGPPC
medium	medium	**PP**	weak	medium	FEPPC
medium	weak	**PP**	weak	weak	FCPPC
weak	strong	**PP**	weak	weak	SGPPC
weak	medium	**PP**	weak	weak	SEPPC
weak	weak	**PP**	weak	weak	SCPPC
